# *Smart Secondary Metabolites* in Marine Environments: The Case of Elatol

**DOI:** 10.3390/md24020061

**Published:** 2026-02-01

**Authors:** Angélica R. Soares, Nathalia Nocchi, Ana R. Díaz-Marrero, Renato C. Pereira, José J. Fernández

**Affiliations:** 1 Grupo de Produtos Naturais de Organismos Aquáticos (GPNOA), Instituto de Biodiversidade e Sustentabilidade (NUPEM), Universidade Federal do Rio de Janeiro (UFRJ), Av. São José do Barreto 764, São José do Barreto, Macaé 27965-045, Rio de Janeiro, Brazil; 2 Instituto Universitario de Bio-Orgánica Antonio González (IUBO AG), Universidad de La Laguna (ULL), Avenida Astrofísico Francisco Sánchez 2, 38206 La Laguna, Tenerife, Spain; nathalianocchi@ull.edu.es; 3 Biotecnología Marina, IUBO-ULL, Unidad Asociada al IPNA-CSIC, 38206 La Laguna, Tenerife, Spain; 4 Servicio General de Apoyo a la Investigación (SEGAI), Universidad de La Laguna, Avenida Astrofísico Francisco Sánchez s/n, 38200 La Laguna, Tenerife, Spain; 5 Instituto de Productos Naturales y Agrobiología (IPNA), Consejo Superior de Investigaciones Científicas (CSIC), Avenida Astrofísico Francisco Sánchez 3, 38206 La Laguna, Tenerife, Spain; 6 Departament of Marine Biology, Biology Institute, Federal Fluminense University—UFF, Niterói 21941-590, Rio de Janeiro, Brazil; rcrespo@id.uff.br; 7 Departamento de Química Orgánica, Universidad de La Laguna (ULL), Avenida Astrofísico Francisco Sánchez s/n, 38203 La Laguna, Tenerife, Spain

**Keywords:** *Smart Secondary Metabolite*, (+)-elatol, (−)-elatol, marine chamigrene, marine sesquiterpene, marine natural product

## Abstract

The concept of “*Smart Secondary Metabolites*” is introduced here to describe a privileged class of natural products defined by structural originality, biosynthetic adaptability, and broad interaction potential with biological systems. Elatol, a halogenated sesquiterpene chiefly produced by *Laurencia* red seaweeds and occasionally accumulated by their consumers, exemplifies this concept with remarkable clarity. Its biosynthesis unfolds from farnesyl diphosphate via *γ*-bisabolane cations, bromochlorination, and stereoselective cyclization to chamigrene scaffolds, generating both (+)- and (–)-enantiomers, two metabolites with clearly distinct potential ecological roles and pharmacological profiles. This review synthesizes the current state of knowledge on elatol’s distribution, biosynthetic origins, ecological relevance, and therapeutic potential. Elatol serves as a multifunctional chemical mediator, fulfilling defensive, communicative, and regulatory roles whose intensity shifts in response to herbivory, biofouling, temperature, and salinity. In parallel, its potent activities against infectious, metabolic, and neoplastic diseases highlight its growing value as a drug lead, reflected in a rising number of patent claims. Altogether, elatol emerges as a model *Smart Secondary Metabolite* whose ecological sophistication and biochemical versatility position it as a promising scaffold for marine-derived drug discovery.

## 1. Introduction

Approximately 748,548 natural products are currently known [1], of which only 5.8%, about 43,705 compounds, are from marine organisms [2]. Although this proportion is still not very representative, over recent decades, the number of metabolites isolated from marine species has significantly increased, and many compounds have been reported [3]. To cope with a wide range of habitats and different growing conditions (e.g., salinity, pressure, tide influences, reduced sunlight, herbivore pressure), these organisms have developed diverse secondary metabolic pathways that generate a vast number of unusual chemical compounds to accommodate their diverse lifestyles [4].

Natural products from seaweeds have been widely explored, since the beginning of civilization, for human use as food and for medical treatments, starting with the traditional knowledge of tribes and ethnic groups [5]. Many chemicals from seaweeds have economic importance and are broadly used [6]. Recent trends in drug research from natural sources have indicated that these organisms are a promising source of novel biochemically active compounds [7]. Seaweeds survive in a competitive environment and, therefore, have developed defense strategies that have resulted in a significant level of chemical structural diversity in various metabolic pathways and ecological functions [8].

The specialized chemical machinery of these organisms has yielded a series of natural compounds with unique characteristics, which are defined here, for the first time, as *Smart Secondary Metabolites*. *Smart Secondary Metabolites* are a privileged class of natural compounds that are characterized by the simultaneous combination of aspects. Those are the following:

- *A singular chemical structure*: These compounds usually have unique and well-defined molecular architectures, which are often complex and stereochemically rich.
- *A high metabolic production*: Although secondary metabolites are not essential for basic cellular survival, these molecules are produced in significant quantities. They may be biogenetic precursors of structurally related compounds.
- *Potent biological activities*: These secondary metabolites have shown strong effects on diverse biological systems (e.g., antimicrobial, anticancer, anti-inflammatory).
- *The ability to interact with different biological targets*: They often have promiscuous or highly specific binding capabilities.
- *A significant ecological or chemotaxonomic value*: These compounds play ecological roles and are useful in classifying species. They may be biogenetic precursors of structurally related compounds.

Red seaweeds of the genus *Laurencia* J.V. Lamouroux (Rhodophyta, Ceramiales, Rhodomelaceae) comprise around 141 species that can be found in littoral and sublittoral habitats, at depths up to 65 m, in temperate and tropical regions of all oceans [9]. *Laurencia* species together with the genera *Laurencia*, *Osmundea*, *Chondrophycus*, *Palisada*, *Yuzurua*, and *Laurenciella* form the group called the “*Laurencia* complex” [10]. *Laurencia* species produce an amazing array of natural products, and they are widely recognized as one of the richest sources of new secondary metabolites [11], many of which exhibit relevant ecological roles (e.g., Pereira et al. [12]) and diverse biological activities (e.g., Machado et al. [13]). Among them, sesquiterpenes are the most abundant group of natural products isolated from species of the genus *Laurencia*. They are C_15_ terpene compounds consisting of three isoprene units with a high degree of halogenation and a wide range of skeleton types, including chamigrane-type [11,14,15].

**Figure 1:**
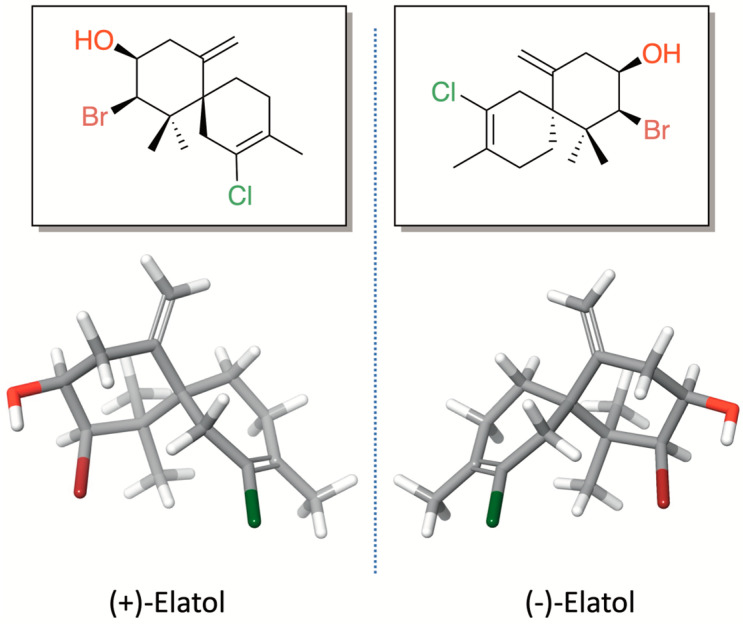
Chemical structures of (+)-elatol and (−)-elatol.

Elatol (2-Bromo-8-chloro-1,1,9-trimethyl-5-methylenespiro[5.5]undec-8-en-3-ol) (Figure 1) is a pale yellow oil with the molecular formula C_15_H_22_OBrCl. This compound was named in honor of the seaweed species from which it was first isolated, *L. elata,* on the coast of New South Wales, Australia [16]. The first studies on elatol were primarily focused on its chemical characterization and on developing synthetic approaches for its production [17,18,19,20,21,22,23,24]. This chamigrane-type sesquiterpene is characterized by a spiro [5.5] undecane skeleton bearing a stereogenic quaternary carbon at the spirocycle junction (C-6). The first six-membered ring (ring A), which is densely functionalized, contains three stereocenters, including two geminal methyl groups bonded to the same quaternary carbon atom, as well as a bromine and a hydroxyl group joined to two distinct methyl carbons and an exocyclic methylene on an olefinic quaternary carbon. On the other hand, the second six-membered ring (ring B) features a methyl group and a chlorine atom, each attached to quaternary unsaturated carbons.

**Figure 2:**
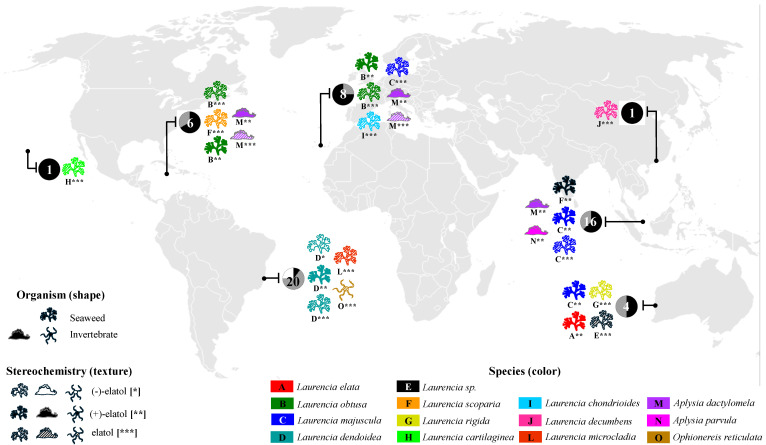
Global distribution of elatol obtained from *Laurencia* species and marine invertebrates, indicating the organismal source (seaweed or invertebrate) and its stereochemistry (represented by texture and asterisks: no fill and [*] = (−)-elatol, solid fill and [**] = (+)-elatol hatched pattern, and [***] = elatol—enantiomer not determined). Each circle represents the proportion of each enantiomer isolated in a region ((−)-elatol = white, (+)-elatol = black, elatol—enantiomer not determined = gray). The numbers within the circles correspond to the total number of reported locations of elatol isolation, while the colors and letters identify the *Laurencia* or the marine invertebrate species responsible for its production.

Elatol is widely found in several species and populations of the genus *Laurencia* worldwide—*L. elata* [16], *L. obtusa* [17], *L. pacifica* [25], *L. majuscula* [22], *L. scoparia* [23], *L. rigida* [26], *L. cartilaginea* [27], *L. chondrioides* [28], *L. decumbens* [29], *L. mariannensis* [30], *L. microcladia* [31], *L. similis* [32], and *L. obtusa* (now *L. dendroidea*) [33]—in different regions of the world (Figure 2 and Table S1, Supplementary Materials). Elatol has also been found in some marine invertebrates, such as sea hares (e.g., Schmitz et al. [34]; Wessels et al. [35]; Vairappan et al. [36]; Palaniveloo & Vairappan, [37]), and in the brittle star *Ophionereis reticulata* [38] (Figure 2 and Table S2, Supplementary Materials). The first report of elatol in marine invertebrates was in 1982 from *Aplysia dactylomela* [34]. All these invertebrates are well-known specialized feeders of chemically defended algae, such as *Laurencia* species and other seaweeds, and can accumulate and use their compounds for their own benefit, for example, as part of their defense strategy [39].

Given its widespread occurrence across multiple *Laurencia* species and its presence in various marine invertebrates, elatol emerges as a metabolite of particular ecological and chemical relevance within marine systems. Its consistent detection in taxonomically and geographically distinct organisms underscores its importance and has motivated extensive investigation over the past decades. In this context, elatol stands out as one of the most representative and well-studied secondary metabolites associated with *Laurencia.*

Previous and exciting reviews have examined the *Laurencia* chemistry, focusing on the genus’s remarkable richness and chemical diversity, as well as the bioactivity of its metabolites [15,40,41,42,43,44,45]. Here, we provide a detailed and comprehensive review of the current knowledge on elatol, one of the most studied and relevant metabolites produced by *Laurencia*. This review offers a comprehensive overview of the occurrence, biosynthesis, ecological roles, biological activities, and biotechnological potential of elatol.

## 2. Search Strategy for Studies on Elatol

This review was conducted in accordance with the PRISMA 2020 guidelines (*Preferred Reporting Items for Systematic Reviews and Meta-Analyses* [46]) to provide a descriptive and narrative overview of the available studies on elatol. A comprehensive literature search was performed using the Web of Science (WoS), ScienceDirect, and SciFinder-n databases, covering publications from 1974 to 2025. Searches were last updated in November 2025.

The search strategy was based on the keyword “*elatol*”, applied across all searchable fields. No Boolean operators or truncations were used in order to maintain a specific strategy focused on the target compound. Eligible studies were peer-reviewed journal articles published in English in which elatol from natural sources was investigated in taxonomical, ecological, and/or pharmacological–biological contexts, or studies describing its isolation and chemical characterization, including analytical or structural data.

To ensure consistency and comparability of the data, the review was restricted to peer-reviewed research articles, while other document types, including conference abstracts, books, book chapters, editorials, and technical reports, were not included. Patent searches were conducted using the Lens.org and Espacenet databases, with no temporal restriction. A detailed description of the search procedures and the workflow used for study selection is provided in the Supplementary Materials.

The comprehensive search identified 241 records across the selected databases: WoS (*n* = 75), ScienceDirect (*n* = 93), and SciFinder-n (*n* = 73). After excluding 58 records during the title and abstract screening phase and removing 53 duplicates, 130 records remained for full-text eligibility assessment. Of these, 50 studies were excluded for not meeting the minimum eligibility criteria. Ultimately, 80 studies were included in this review (Figure S1 and reference list in the Supplementary Materials).

## 3. Biogenesis and Metabolic Production of (+) and (–)-Elatol

Until 2011, only (+)-elatol was known. Then, the enantiomer (−)-elatol was reported for the first time from a population of *L. dendroidea* collected on the Brazilian coast [47]. Since then, the (+)-isoform and (−)-isoform of elatol have been described in different *Laurencia* populations along the Brazilian coast [43,48,49,50,51,52,53,54]. Given this unusual enantiomeric and isoform diversity, *L. dendroidea* seems to be a particular case in marine natural product chemistry. Enantiomeric compounds can be found in different genera or species, where one enantiomer is isolated from one species while the other enantiomer is isolated from another different species or genera. In addition, enantiomers may also be isolated as either a racemic or scalemic mixture (where one enantiomer predominates) from the same individual [55,56]. Thus, the elatol enantiomer production appears to be related to genetic and/or phenotypic plasticity, because only one of the enantiomers is exclusively produced depending on the population.

It is common to find studies about the biological activity of marine natural products without the full chemical identification of a metabolite. This becomes even more common for substances with one or more chiral centers. Data on the stereochemistry of a compound are even rarer, especially when it is a known compound. There are countless examples, especially older ones, of a potential activity without identifying the stereoisomer under investigation. The absence of this information hinders knowledge about the activity of a substance and slows advances in the development of medicines from marine origin. This fact becomes even more critical when it comes to enantiomers. Each enantiomer of a given chiral drug may have its own particular pharmacologic profile, differing significantly in its pharmacodynamics, pharmacokinetic characteristics, and toxicity mechanisms [57,58].

### 3.1. Biosynthetical Pathway

**Figure 3:**
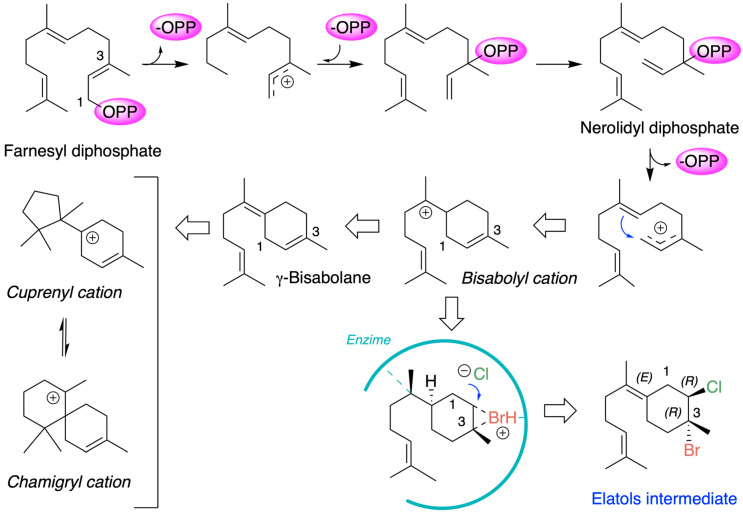
Biosynthetic scheme of chamigrane metabolites.

Chamigrane metabolites are produced through the terpenoid biosynthetic pathways that originate from the common precursor farnesyl diphosphate (FPP). The generally accepted mechanism for the formation of chamigrene metabolites involves the generation of a *γ*-bisabolane intermediate, which in turn leads to the formation of the cuprenyl cation, or directly to the chamigryl cation, either through rearrangement or through a ring expansion of the cuprenyl cation (Figure 3) [59,60].

Chamigrenes are frequently co-isolated with cuprenenes and trichodiene from various terrestrial plant species. Halogenated chamigrene derivatives have also been obtained from *Laurencia* species, often together with laurane-type sesquiterpenes. In contrast, chamigrenes have occasionally been isolated in the absence of other related sesquiterpenes [44]. From a biosynthetic perspective, this latter case can be explained by the protonation of farnesyl pyrophosphate followed by the loss of pyrophosphate, yielding a γ-bisabolane intermediate. The co-occurrence of both (–)- and (+)-chamigrene in *Laurencia* species is particularly unique among marine organisms. Within this group of compounds, the biosynthesis of elatol can be rationalized by assuming that the enzymatic addition of bromochloride occurs at the bisabolonium ion stage, prior to cyclization into the chamigrene framework.

**Figure 4:**
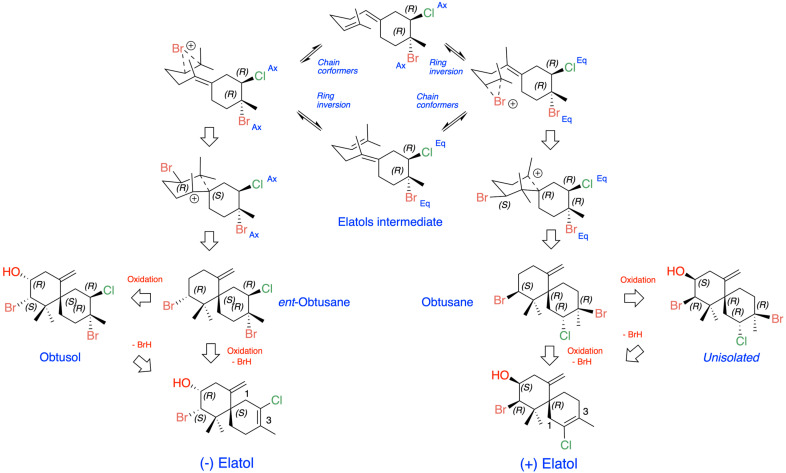
Biosynthetic scheme proposed for (+) and (−)-elatol.

The origin of (+)- and (−)-elatol can be rationalized as shown in Figure 4. At the enzyme active site, the bisabolonium ion can undergo bromination accompanied by the nucleophilic incorporation of a chloride anion at carbon C-2, generating a *trans*-diaxial bromochloride intermediate. This species can then equilibrate to the more stable *trans*-diequatorial system. The subsequent deprotonation at carbon C-6 yields the corresponding γ-bisabolane intermediates, which serve as precursors of both (+)- and (−)-elatol. A bromo-induced carbocyclization on both the *α*- and *β*-face of these intermediates leads to the formation of the respective chamigrene intermediates. Finally, through *syn*-dehydrobromination, followed by enantiospecific oxidation at carbon C-9, this pathway affords (+)- and (−)-elatol [17,40,61]. This proposed biosynthetic pathway is further supported by the isolation of structurally related metabolites such as obtusane, *ent*-obtusane, and obtusol.

### 3.2. Intra-Cellular Storage Vesicles and Transport of Elatol to Cell Surface

Since 1980, it has been suggested that refractory structures called *corps en cerise* (*CC*) would be involved in the storage of halogenated metabolites in the red seaweed *L. snyderae*, based on the presence of a simple bromine secondary emission peak [62]. *Corps en cerise* are spherical, electron-dense subcellular inclusions characteristic of *Laurencia* species that resemble small cherries under electron microscopy and are important as sites linked to the production, storage, and transport of halogenated secondary metabolites involved in chemical defense, as well as useful taxonomic markers [62,63]. However, it was not until almost 30 years after this evidence that *CC* were isolated from *L. obtusa* (now *L. dendroidea*) cells and X-ray diffraction detected bromine and chlorine from the elatol sesquiterpene and confirmed the presence of this compound inside these vesicles [63]. Additional observations from fluorescence optical microscopy indicated the localization of halogenated metabolites in smaller *CC* vesicles also distributed in the cytoplasm of *L. dendroidea* cells [63].

The presence of stalk-like structures connecting the *CC* to the cell periphery of *L. dendroidea* was also evidenced, enabling the transport of elatol, as well as other chemicals, from inside the *CC* to the surface of *L. dendroidea* [63]. Microfilaments found within these connections were verified as essential structures facilitating vesicle traffic containing these chemicals to the cell surface [64]. Recently, the participation of ABC proteins was evidenced as a key mechanism regulating elatol transport to the surface of *L. dendroidea* cells and the defensive system of this seaweed against herbivory and fouling [65].

Since *CC* were located at, or close to, the cell surface and exhibited a connection through channels to the surface cell layer, this evidence about storage and exudation mechanisms is essential for understanding the previously reported ecological roles of elatol as a defense against herbivory [12] and fouling [12,33]. It is also possible to hypothesize that this must be the mechanism employed by *Laurencia* species that have *CC* and are chemically defended against these natural enemies.

The demonstration of the intricate cellular machinery involved in the biosynthetic pathways for terpene production, as well as how these chemicals are stored in *CC* and regulated during exocytosis, still lacks complete evidence regarding the proteins involved in their transmembrane transport in marine macroalgae. Recently, elatol was used in experimental approaches at the biochemical and cellular levels to investigate the localization of ABC protein transporters in *L. dendroidea* and their role in the transport/accumulation of secondary metabolites and the defensive mechanisms of seaweeds against fouling and herbivory [65].

Altogether, the current knowledge suggests that the combined action of *CC* vesicles and ABC transporters enables the precise delivery of elatol to the algal surface, thereby ensuring an effective defensive barrier against herbivores and foulers, while underscoring the sophistication of chemical defense mechanisms in *Laurencia*.

## 4. Ecological Roles

Elatol is one of the best-studied marine secondary metabolites regarding its ecological roles. Over the past four decades, a combination of several field and laboratory studies have extensively investigated the ecological roles of elatol, and evidence suggests that elatol mediates competitive interactions as an allelopathic agent and defends against diverse groups of consumers, pathogens, and fouling. It also mentions exciting examples of elatol acting as a chemical cue in the interactions of *Laurencia* with its associated specialist herbivores. Spatial and temporal variations, in addition to the storage, transport, and exocytosis of this substance, have also been demonstrated. Although the stereochemistry of elatol is often not identified in ecological studies, complicating the comparison of bioassay results and the assessment of its activity variability, the available evidence on these processes is critically reviewed and discussed. This highlights the diverse ecological roles that elatol may fulfill within marine ecosystems, thereby enhancing the adaptive significance of this metabolite for several algae of the genus *Laurencia*.

### 4.1. Variability of Elatol Contents

Variability in secondary metabolite amounts is a crucial aspect for chemically mediated interactions to be established and for the understanding of the ecological and evolutionary aspects of these interactions [66]. Evidence on the variability in elatol contents was found in *L. dendroidea* in different scales and contexts, from cellular to populational, due to intrinsic storage procedures and transportation mechanisms or extrinsic environmental variables.

The first evidence of a broad spectrum in the intra-populational and intra-thallus variations of elatol contents was obtained from the analysis of individuals (*n* = 70 for intra-thallus; *n* = 65 for surface analysis) from a single population of *L. dendroidea*. The concentration within-thallus was higher (9.89 mg·g/L algal dry weight, dw) than the amount found at the surface of the *L. dendroidea* (0.0059 mg·g/L dw or 0.5 to 10.0 ng·cm^2^ dw) [67]. This difference represents amounts more than 1600 times higher within-thallus, compared to lower values found on the thallus surface.

Broad-spectrum assessments have also been carried out comparing populations from different locations on the Brazilian coast. Samples of *L. dendroidea* from six distinct locations covering a range of more than 600 km along the Brazilian coast, from Manguinhos beach (20°11′13.9″ S, 040°11′25.4″ W) to Vermelha beach (23°11′35.0″ S, 044°38′39.0″ W), found different amounts or even an absence of elatol [68]. Producer specimens of *L. dendroidea* from three localities had between a 23 and 52% area of compound peaks obtained through gas chromatography coupled with mass spectroscopy analysis [68].

**Figure 5:**
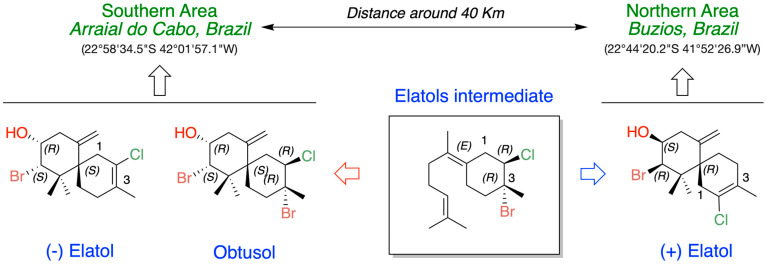
Differentiation in the metabolic production of elatols and obtusol in *Laurencia dendroidea* collected in two areas of the Brazilian coast.

In the context of intra- and inter-populational variability, a broad range of variation was observed in the content of elatol in *L. dendroidea* from four distinct locations, with distances ranging from ca. 300 to 1800 km along the Brazilian coast (Figure 5). Elatol concentrations exhibited a 2- to 10-fold variation among *L. dendroidea* individuals within the same locality, whereas comparisons among the four sampled localities revealed approximately three orders of magnitude of variation, with values spanning from 0.001% to 1.24% of dry biomass [66].

Knowledge on how environmental and genetic factors promote phenotypic plasticity in the production of chemical defenses in seaweeds is still unclear. However, bioassays conducted under common garden laboratory conditions showed that the same relationship was maintained between the different elatol levels found in specimens from the environment [66,69]. Thus, the variation in elatol production in populations of *L. dendroidea* from four different locations appears to be due to phenotypic plasticity or local adaptation and may be under genetic control [69].

Clones were maintained under constant conditions of temperature, salinity, nutrient availability, and irradiance to verify the internal regulation of physiological process, specifically the correlation between the production of elatol and the activity of photosystem II–φPSII. A presumed genetic basis is the endogenous rate of daily variation in elatol production in this seaweed, which is inversely correlated with the quantum yield of photosystem II–φPSII [70].

In addition to being conditioned by intrinsic aspects of *L. dendroidea*, elatol levels can also vary depending on environmental conditions or variables. For example, the levels of elatol in *L. dendroidea* were clearly influenced by temperature and salinity, since severe conditions of these parameters under laboratory conditions promoted a decrease in this chemical [71]. This variability can result in the differential susceptibility of *L. dendroidea* specimens to natural enemies, since elatol is a known defensive chemical in *Laurencia* species (e.g., Pereira et al. [12]).

### 4.2. Defense Against Herbivory

The defensive properties of elatol were initially hypothesized based on studies of the toxicity exhibited by this sesquiterpene, as well as field observations in which it was found that *L. obtusa*, which produces it (more than 3% dry weight), is not consumed by herbivores [72]. Further studies experimentally confirmed the defensive effects of elatol against herbivores, such as the gastropods *Littorina striata* and *Osilinus atratus* [73], the sea urchin *Diadema antillarum* and reef fishes [74], and the crab *Pachygrapsus transversus* and the sea-urchin *Lytechinus variegatus* [12]. Contrary to the examples of the defensive action of elatol mentioned above, this compound obtained from *L. microcladia* did not inhibit consumption by the urchin *Echinometra lucunter*, which is directly associated with this seaweed in the natural environment [31].

These conflicting results highlight the challenges of comparing the defensive activities of a compound in the absence of comprehensive chemical characterization, particularly for chiral molecules, since none of these studies clearly identified which isomer was used. Nevertheless, it can be assumed that the ecological role of elatol is likely widespread in the marine environment, as evidenced by its other defensive functions, such as antifouling [75] and allelopathic effects [69].

These observations suggest that elatol functions as an important chemical defense in marine ecosystems, with effectiveness that may depend on the target herbivore species, the specific *Laurencia* source, and the isomeric form, highlighting its broader ecological significance beyond herbivory.

### 4.3. Antifouling Properties

Antifouling is an activity that has long been demonstrated for elatol, which is of ecological and biotechnological interest, and which in this review will be approached as an eminently distinct functionality. Thus, for an ecological approach, it is essential to know the levels of elatol on the surface of the organism that produces it, whereas a biotechnological approach uses, for example, the total amount of elatol found in the organism or concentrations practiced in bioassay protocols. However, conducting bioassays with natural concentrations of chemicals was [76], and still is, one of the great challenges in marine chemical ecology, as this information is rarely available, although a precise method has been developed for quantifying substances on the surface of macroalgae [77] and in CC vesicles [67].

Using a protocol developed for the precise quantification of elatol on the surface of *L. obtusa* (now *L. dendroidea*) [67], the ecological functionality of elatol as an antifouling agent was accurately assessed, i.e., using natural concentrations of this metabolite found on the surface of *L. dendroidea* [78]. Although no antifouling activity was found in this study, this does not preclude its activity, since the concentration of elatol is dynamic as a function of natural fluctuation [78], and studies with higher concentrations than the one tested are unequivocal about this property of this metabolite [33]. In addition, the increase in the concentration of elatol on the surface of *Laurencia* may be promoted by the presence of epibionts [75]. This is consistent with the observation that the traffic of secondary metabolites inside vesicles (*CC*) could allow for an increased surface concentration of the metabolite without causing toxicity for *L. dendroidea* cells, while also permitting the regulation of metabolite exudation [63,70].

Elatol is also a well-known metabolite that has been characterized as a broad-spectrum antifouling agent in applied or biotechnological contexts, based on tests carried out on a wide variety of marine fouling organisms. One of the first findings showed that elatol exhibited a strong effect against marine bacteria *Vibrio fischeri* and inhibited the settlement of larvae of the balanid *Balanus amphitrite* and the bryozoan *Bugula neritina* [79]. Subsequent studies have confirmed elatol as a potent antifouling agent and broadened its spectrum of action, since this sesquiterpene exhibited a pronounced antifouling activity, also evidenced in field studies [33].

The high and promising antifouling potential of elatol has promoted initiatives for a molecular approach aimed at understanding the biosynthetic pathways that lead to the production of this sesquiterpene [80,81], or even heterologous cloning to obtain this and other chemicals of biotechnological interest produced by *L. dendroidea* [82]. Given the strong antifouling properties of elatol, additional studies are urgently needed to develop technologies that can produce this compound in sufficient quantities for further bioassays, as well as to meet the demands of a potential future market.

### 4.4. Allelopathy

Allelopathy is a strategy employed by various benthic marine organisms and may play significant roles in structuring benthic communities, since it can exert effects on the patterns of spatial distribution of competing organisms (e.g., Ribeiro et al. [83]). The occurrence of allelopathy in algae, as well as the mechanisms underlying this phenomenon, has been little studied, but a recent review brings together the current knowledge, opportunities, and challenges in this emerging field [84]. Antifoulants can also be considered allelopathic, but have been addressed separately in this review. Elatol has also been evidenced as a strong allelopathic agent, which represents an ecological strategy employed by *Laurencia* species that produce and release this toxic metabolite into the environment to increase their competitive ability.

An evaluation of the autotoxicity (intraspecific allelopathy) effects of elatol from *L. dendroidea* was investigated by Sudatti et al. [69] through experiments under laboratory conditions, using chlorophyll fluorescence imaging to measure the inhibition of photosynthesis (PSII) as a variable response. Individuals of *L. dendroidea* from Azeda and Forno beach on the southern coast of Brazil were inhibited by (+)-elatol, with IC_50_ = 87 µg/mL and 277 µg/mL, respectively, demonstrating autotoxicity for both chemotypes. Thus, a higher density can reduce the growth of seaweeds, and autotoxicity may play an important role in structuring seaweed *L. dendroidea* through density control [69]. These findings also broaden the understanding of the adaptive and ecological roles of elatol and confirm that its storage inside CC is essential to prevent autotoxicity.

### 4.5. Chemical Cue

Various aquatic organisms rely on chemical compounds emitted by other organisms, which serve as signals or cues, to locate and select food, choose partners and habitats, and detect imminent threats such as predation [85,86].

The close relationship between *Alpysia* and *Laurencia* has long been known, since these mollusks selectively feed on species of this macroalgae (e.g., Pennings et al. [39]). For example, elatol from *Laurencia* species was found in *A. dactylomela*, supporting the chemical relationship between them [37]. Through an experimental approach, it has been shown that specimens of *A. brasiliana* are attracted to the (+)-elatol produced as the major compound in *L. dendroidea* [52]. In addition to supporting the close relationship known in the literature for many years, this chemical mediation is presumably of great relevance to young *A. brasiliana*, since living inside *L. dendroidea* minimizes the risk of being consumed.

The study on *A. brasiliana* revealed that chemical cues released by this specialist herbivore can induce the production and accumulation of defensive metabolites, such as elatol, in *L. dendroidea* [87]. This chemically mediated interaction demonstrates that seaweeds can detect herbivore-associated cues and respond adaptively by upregulating their chemical defenses. Such findings highlight the ecological importance of infochemicals in shaping seaweed–herbivore dynamics, reinforcing that defense expression in *Laurencia* is not static but context-dependent, environmentally triggered, and integrated with the biosynthetic, cellular, and transport mechanisms discussed in this article.

These observations underscore the pivotal role of chemical cues, such as elatol, in structuring ecological interactions between *Laurencia* species and *Aplysia* and influencing feeding behavior, habitat selection, and survival, and highlight the evolutionary significance of chemically mediated relationships in marine environments.

### 4.6. Elatol as Chemical Marker in Marine Food Web

Chemical mediation regulates behavioral interactions between species, and in predator–prey relationships, chemical compounds can be food web markers, which is particularly important in the context of seaweed and marine mollusks [88]. For example, several sea hares (Opistobranchia: Anspidae) and gastropod mollusks worldwide have developed chemically mediated relationships with seaweeds, particularly those from coiled, reduced, or absent shell species [89]. For example, sea hares from the widespread herbivore genus *Aplysia* are known to feed on *Laurencia* species and concentrate chemicals from these red seaweeds in their digestive glands [36,90].

A recent review highlighted that the interaction between the species *A. dactylomela* and its seaweed diet can provide information on the biogeography and diversity of seaweeds, the use of bioaccumulated chemicals for its own defense mechanism, and their ecological roles in the marine environment [91]. Due to the multiple ecological functions exhibited by elatol, it is reasonable to infer its importance for Anaspidae species that accumulate it from their *Laurencia*-based diet.

Elatol has also been isolated with marine invertebrate species (Table S2, Supplementary Materials) such as the sea hare *A. dactylomela*, first in Puerto Rico [34] and later in specimens of this mollusk also from locations in Puerto Rico [92,93], as well as from different locations in the Canary Islands [35,94,95]. In addition, elatol has also been isolated from another species of sea hare, *A. parvula* [36], and from the brittle star *Ophionereis reticulata* [38]. Among these studies, only Schmitz et al. [34] reported stereochemical data, identifying the (+)-isoform of elatol.

These studies had different objectives, such as the isolation and structural elucidation of elatol [34,38,92,93,95], as well as evaluations of some of its biological activities [35,37,94]. Notably, studies have demonstrated that elatol is diet-derived in these mollusks, specifically from *Laurencia* species [36,37]. Elatol found in the ophiuroid *O. reticulata* may also be derived from its diet of *Laurencia* species. Although this species is omnivorous, it shows a preference for green and red macroalgae [96]. While only fragments of red algae were identified [96], it is presumed that *Laurencia* species were among them.

These few examples show that elatol may be a food web marker and could, in the future, help establish precise trophic relationships between sea hare species and *Laurencia*, as well as highlight the broad biogeographic ecological roles of this sesquiterpenoid compound.

## 5. Pharmacological Properties of Elatol

Throughout evolution, benthic or sessile organisms have developed complex and unique biochemical defense mechanisms that ensure their survival and perpetuation in highly competitive environments or under unique abiotic conditions. Many of these mechanisms involve the production of substances that act against predators, fouling organisms, and pathogens. Some of these bioactive compounds have shown a promising potential for the treatment of various diseases, including infectious diseases, metabolic disorders, and cancer. These discoveries are particularly relevant in the context of increasing drug resistance and the demand for new therapies. The exploration of these compounds not only offers new perspectives for pharmacology but also contributes to biotechnological innovation and the development of more effective and selective drugs.

The first evidence of the pharmacological potential of elatol was shown by Norris and Fenical in 1982 [72], when the inhibition of the cell development of fertilized sea urchin eggs and antibacterial human pathogens was demonstrated. Since then, the increasing interest in marine natural product drug discovery in recent decades has boosted investigations of elatol’s bioactivities, highlighted in studies on its anticancer, antimicrobial, antiparasitic, anti-colinesterase, acaricidal, and insecticidal bioactivities. However, the majority of these studies do not provide information about the stereochemistry of elatol, which complicates data comparisons and the standardization of tests. Understanding elatol as a bioactive molecule of pharmacological interest provides new perspectives for the development of innovative treatments against various diseases. Below, the biological activities of elatol reported in the literature are presented in a descriptive, narrative manner, with the aim to compile and synthesize the principal experimental findings across different biological models.

### 5.1. Antiproliferative Potential

In 1997, Juagdan et al. [27] evaluated, for the first time, the anticancer potential of elatol. Although its stereochemical data were not reported, the cytotoxic activity of elatol and three other sesquiterpenes isolated from *L. cartilaginea* against four cancer cell lines was demonstrated. An IC_50_ of 1.0 μg/mL for murine lymphocytic leukemia (P-388) and the same IC_50_ of 0.1 μg/mL for cell lung carcinoma epithelial (A-549), human colon carcinoma (HT-29), and human melanoma (MEL-28) were obtained. This pioneering work not only established the foundation for considering elatol as a promising anticancer agent, but also motivated subsequent studies investigating its mechanism of action, particularly its modulation of translational control and selective cytotoxicity toward different tumor types.

An unidentified isoform of elatol isolated from *L. microcladia* was investigated using in vitro and in vivo experiments. In an in vitro bioassay, the compound reduced, in order of decreasing sensitivity, the cell viability of murine fibroblast (L929), human prostate carcinoma (DU145), human mammary adeno-carcinoma (MCF-7), A549 (human lung carcinoma), and murine melanoma B16F10 cells [97]. These findings demonstrate elatol’s selective cytotoxicity across multiple cell lines, supporting its potential for further preclinical development as a targeted anticancer agent.

In a study of chemically modified elatol and other *Laurencia* sesquiterpenes, the in vitro cytotoxic effects against lung carcinoma A549 and embryo rhabdomyosarcome (RD) tumor cells were evaluated. Elatol was at least twice as active as its derivatives in the two strains tested [98], highlighting the critical role of its natural structure for anticancer potency.

The (+)-isoform of elatol and eight other chamigranes were isolated from *L. majuscula*. (+) Elatol demonstrated a strong cytotoxic activity against human cervical epithelioid carcinoma (HeLa), human breast carcinoma (MCF-7), and murine lymphocytic leukemia (P-388), with an IC_50_ ≤ 1.0 μg/mL [99]. The same isomer was also isolated from *A. dactylomela*, a specialized herbivory of the *Laurencia* species collected from Tenerife Island and tested against gastric carcinoma (HM02), liver carcinoma (Hep G2), and breast carcinoma (MCF 7); the authors showed an IC_50_ < 1.0 µg/mL for all cancer cells [35]. From another population of *A. dactylomela* collected from La Palma Island, Dias et al. [94] isolated this sesquiterpene and tested it against human carcinoma of the cervix (HeLa), human cervical carcinoma (Hep-2), and African green monkey kidney (Vero). The compound was active against all cell lines, with an IC_50_ varying between 1.3 µM (HeLa) and 25.0 µM (Vero). These results collectively underscore the broad-spectrum cytotoxic potential of the (+)-elatol isoform across multiple *Laurencia* species and even in their specialized consumers, highlighting both the consistency of its bioactivity and the critical role of its stereochemistry for anticancer effects. Such reproducible potency across diverse sources and cell lines reinforces the promise of (+)-elatol as a lead compound for further preclinical investigation.

Six halogenated sesquiterpenes isolated from *L. dendroidea* were tested in vitro against four drug-resistant cancer cell lines. Among all cell lines, (−)-elatol showed the most promising results against colon cancer cells (Colo-205), with an IC_50_ of less than 10 μg/mL, inducing apoptosis through the activation of caspases 2, 4, 6, and 8. For resistant murine melanoma cells (B16F10), an IC_50_ of 54.65 ± 4.74 μg/mL was observed. Additionally, IC_50_ values ranging from 30 to 40 μg/mL were reported for human lymphoma (U937) and acute T-cell leukemia (Jurkat). Importantly, this enantiomer demonstrated no cytotoxicity towards peripheral blood mononuclear cells (PBMCs), showing a selective toxicity only towards tumor cell lines [51]. These results underscore the selective cytotoxicity of (−)-elatol toward tumor cells, sparing normal PBMCs, and highlight its apoptotic mechanism via multi-caspase activation, reinforcing its potential as a targeted anticancer agent.

Elatol, from *L. microcladia*, has been shown to act as a potent inhibitor of mitochondrial protein synthesis, exhibiting a strong antileukemic activity [100,101]. The compound selectively suppresses mitochondrial, but not cytoplasmic, translation in chronic myelogenous and lymphoblastic leukemia cell lines, and activates the mitochondrial stress pathway OMA1-DELE1-ATF4, leading to a reduced glycolytic capacity, ATP depletion, and apoptosis. In these studies, elatol demonstrated 10–40 times greater potency than the reference drug, suggesting that it targets mitochondrial metabolism and represents a promising strategy against therapy-resistant leukemias. These findings not only highlight elatol’s exceptional potency and selectivity for mitochondrial translation in leukemia cells but also suggest its potential as a lead compound for developing novel therapies targeting mitochondrial metabolism in treatment-resistant hematological malignancies.

Considering all the evidence, elatol exhibits potent and selective antiproliferative effects across diverse cancer cell lines, including drug-resistant types, through mechanisms such as mitochondrial translation inhibition, apoptosis induction, and metabolic disruption. Enantiomer-specific activity highlights the importance of stereochemistry in its efficacy. While still at the preclinical stage, these findings establish elatol as a promising lead compound for future anticancer therapies.

### 5.2. In Vivo Antitumoral Activity

**Figure 6:**
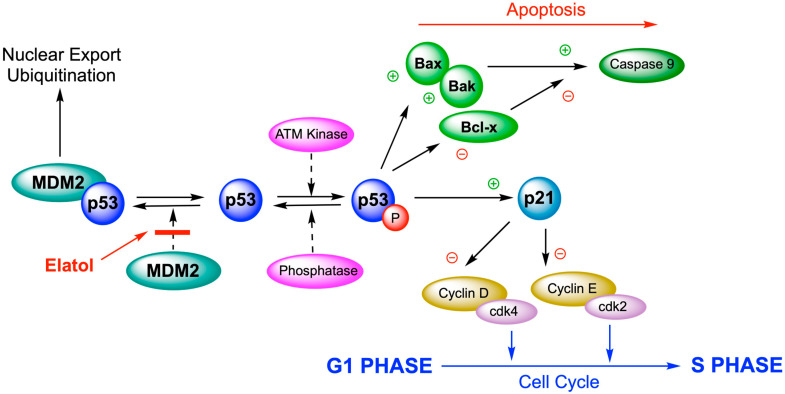
Elatol induces cell cycle arrest in the G1 and the sub-G1 phases, leading cells to apoptosis through the deregulation of p53 proteins.

The antitumor activity of elatol was tested using a murine model for in vivo bioassays; B16F10 cells were used, although they have been less sensitive to in vitro experiments. These cells are a widely used method for assessing in vivo antitumor effects. Elatol reduced cell viability in a time-dependent manner by inducing cell cycle arrest in the G1 and the sub-G1 phases, leading cells to apoptosis. The Western blot analysis of cell cycle proteins demonstrated that elatol reduced the expression of cyclin-D1, cyclin-E, cyclin-dependent kinase (cdk)2, and cdk4. A decrease in bcl-xl and an increase in bak, caspase-9, and p53 expression was also observed. In the in vivo experiment, a treatment with elatol was able to reduce tumor growth in C57Bl6 mice (Figure 6). The volume of primary melanoma tumors in elatol-treated mice (intraperitoneal application 10 mg/kg) was significantly reduced by 71.4% [97].

These results demonstrate that elatol exerts significant antitumor effects both in vitro and in vivo by modulating key cell cycle- and apoptosis-related proteins, supporting its potential as a candidate for further preclinical development in melanoma therapy.

### 5.3. Antiparasitic Properties

The first evidence of the antiparasitic activity of elatol dates back just over a decade. Studies began by evaluating the in vitro antiprotozoal potential of a sesquiterpene isolated from Brazilian *L. dendroidea* against *Trypanosoma cruzi* [102] and *Leishmania amazonensis* [102]. The trypanocidal action of elatol showed a promising dose-dependent potential in anti-Chagas disease, with IC_50_ values of 45.4, 1.38, and 1.01 μM against the epimastigote, trypomastigote. and amastigote *T. cruzi* forms, respectively [102]. In 2012, Desoti et al. [103] showed the mechanism of action of the tripanocid (−)-elatol. The compound caused cell death, inducing the depolarization of the mitochondrial membrane, an increase in the formation of the mitochondrial superoxide anion, a loss of cell membrane and DNA integrity, the formation of autophagic vacuoles, and a decrease in cell volume. Additional studies showed that (−)-elatol leads to the death of the amastigote form of *T. cruzi* through autophagic and apoptotic processes generated by an imbalance of the parasite’s redox metabolism [48]. Collectively, these studies demonstrate that (−)-elatol exerts potent, dose-dependent antiprotozoal effects against *T. cruzi*, acting through mitochondrial dysfunction, oxidative stress, and autophagic/apoptotic pathways, highlighting its potential as a lead compound for anti-Chagas drug development.

The antileishmanial activity was also evaluated, and elatol showed an IC_50_ of 4.0 μM for promastigote and 0.45 μM for amastigote forms of *L. amazonensis* [104]. Elatol showed an effective and specific antiprotozoal inhibition, while at the same time it is not toxic to macrophages [102,104]. Already, in 2011, Machado et al. [47] reported the leishmanicidal activity of (−)-elatol, a new isoform isolated from a Brazilian population of *L. dendroidea*, against *L. amazonensis*. The compound exhibited IC_50_ values of 9.7 ± 1.2 µg/mL and 4.5 ± 0.9 µg/mL for the promastigote and amastigote forms, respectively, activities approximately 7 and 30 times lower than those reported by Santos et al. [104]. However, comparing these results is challenging, especially since the enantiomeric form of elatol used in the latter study was not specified. Furthermore, *L. dendroidea* populations have shown a variable antileishmanial activity, and extracts containing variable concentrations of (−)-elatol may be responsible for this effect [43]. These findings confirm the potent and selective antileishmanial activity of (−)-elatol, while highlighting that efficacy can vary depending on the enantiomeric form and population source, emphasizing the need for careful characterization in future studies.

The (−)-isoform of elatol also exhibited a strong activity against *Schistosoma mansoni* at two different stages of its life cycle. For cercariae, the compound caused 100% mortality at 12.5 µg/mL, while in adult worms it completely inhibited oviposition at 50 µg/mL. Additionally, (−)-elatol demonstrated a potent molluscicidal activity against *Biomphalaria glabrata*, the intermediate host of *S. mansoni*, with 100% mortality observed at 1.56 µg/mL [53]. Schistosomiasis, caused by parasitic trematodes of the genus *Schistosoma*, is a neglected tropical disease that remains a major public health concern in many developing countries [105]. These findings are promising for the development of eco-friendly control strategies, as (−)-elatol could serve as a natural, environmentally sustainable agent for disrupting both the parasite and its intermediate host populations.

The antiparasitic activity of (+)-elatol, its enantiomer (−)-elatol, and nine other chamigrane-type sesquiterpenes isolated from *L. dendroidea* was evaluated against the opportunistic free-living amoeba *N. fowleri*, the causative agent of the rare and often fatal infection known as primary amoebic meningoencephalitis. Among the compounds tested, (+)-elatol exhibited the highest activity against *N. fowleri* trophozoites, with IC_50_ values of 1.08 μM for the ATCC 30808™ strain and 1.14 μM for the ATCC 30215™ strain, both at lower concentrations than the reference drug. Its enantiomer, (−)-elatol, was found to be 34-fold less potent, showing IC_50_ values of 36.77 μM and 38.03 μM, respectively. The markedly higher potency of (+)-elatol compared with its enantiomer and other chamigranes suggests that subtle stereochemical features play a key role in modulating antiparasitic activity against *N. fowleri* [106].

In addition, Chaúque et al. [107] calculated the therapeutic index percentage (TI%) for both enantiomers, revealing a markedly higher TI for (+)-elatol (5441%) compared to (−)-elatol (380%), confirming the superior selectivity and lower cytotoxicity of the (+)-enantiomer. Therefore, both compounds can be considered promising cysticidal and trophocidal drug candidates due to their high potency and low toxicity toward non-target cells, with (+)-elatol standing out as the most selective and safest derivative. The therapeutic potential of (+)-elatol was further confirmed through an in vitro extracellular matrix (ECM)-based adhesion model, where it exhibited a similar or even greater anti-adhesion activity than reference drugs by inhibiting the attachment of *N. fowleri* trophozoites to major ECM proteins across different concentrations and exposure times [108]. The combination of high potency, selectivity, and anti-adhesion activity positions (+)-elatol as a promising candidate for the rational design of new antiparasitic drugs targeting both trophozoite proliferation and host tissue attachment.

Seaweeds are among the most important marine sources of antiparasitic compounds [109,110]. The growing emergence of resistance to existing antiparasitic drugs highlights the urgent need for new bioactive molecules to combat neglected tropical diseases [111]. Within this context, elatol has emerged as one of the promising natural candidates, exhibiting potent, selective, and enantiomer-dependent antiparasitic activity against protozoa, trematodes, and free-living amoebae, acting through mitochondrial dysfunction, oxidative stress, and apoptosis/autophagy pathways. Its high efficacy combined with its low host toxicity underscores its potential as a lead compound for novel antiparasitic drug development.

### 5.4. Insecticidal and Acaricide Activities

Elatol samples isolated from distinct Brazilian *L. dendroidea* populations showed larvicidal activity for the control of vectors of dengue mosquito, *Aedes aegypti*. Elatol showed a moderate larvicidal activity, with an EC_50_ value of 10.7 ppm [112], while (−)-elatol killed approximately 30% of the larvae at 10 ppm [50]. These results underscore the relevance of elatol and its enantiomers as bioactive metabolites with potential applications in vector-control programs, particularly in the search for new larvicides to combat dengue-transmitting mosquitoes.

Elatol also showed acaricidal activity against the mite species *Tetranychus urticae*, a major agricultural pest, causing 40.0 ± 1.6% mortality at a concentration of 2.6 μg/cm^2^ [113], representing an important example of how marine sesquiterpenes can provide promising leads for biopesticides and broaden the biotechnological applications of secondary metabolites from *Laurencia* species.

### 5.5. Antimicrobial Properties

Metabolites from seaweeds are well known for their efficacy against a wide range of pathogens, including multidrug-resistant bacterial strains [114], viruses [115], and opportunistic fungi [116]. These compounds not only offer an alternative to traditional antibiotics and antifungals in a growing scenario of resistance to conventional medicines, but also open up new fronts for the development of more effective therapies with less toxicity in human cells. Many of these antimicrobial activities are attributed to halogenated compounds, such as elatol, found in the red seaweed species Rhodophyta [99].

#### 5.5.1. Antibacterial Activity

A moderate antibacterial activity for elatol isolated from *L. obtusa* was initially demonstrated against human pathogenic bacteria strains [72]. The antibacterial activity of (+)-elatol, isolated from six *L. majuscula* populations [117,118] and *A. parvula* [36] from the Malasya coast, was carried out using marine and human pathological bacteria strains. The sesquiterpene inhibited all 12 marine bacteria tested, with significant antibacterial activities against *Ruminiclostridium cellobioparum*, *Proteus mirabilis*, and *Flavobacterium helmiphilum*, whereas it inhibited 6 of 8 specimens of human pathological bacteria obtained from clinical patients. Elatol was more active against *Staphylococcus epidermis*, *Klebsiella pneumonia*, and *Salmonella* sp. Subsequently, its antimicrobial potential was evaluated in a panel of six strains of bacteria isolated from algae infected with ice-ice disease and six strains of human pathogenic bacteria. Overall, these findings highlight the broad-spectrum antibacterial potential of elatol against both marine and human pathogenic bacteria, indicating its promise as a natural bioactive compound for further pharmacological and biotechnological investigations.

Vairappan et al. [119] demonstrated that elatol showed a highly selective inhibition against seaweed pathogens compared to human pathogens, with an activity comparable or superior to commercial antibiotics. Kamada et al. [120] demonstrated that elatol from *L. majuscula* inhibited three human pathogenic bacteria (*Escherichia coli*, *Salmonella typhi* and *Vibrio cholera*), with MIC and MBC values of 100 μg/mL value 250 μg/mL, respectively. A moderate activity was observed for elatol from *A. dactylomela* against pathogen human bacterial *Bacillus megaterium* [35], whereas elatol isolated from *L. chondrioides*, *L. dendroidea*, and *L. rigida* showed little to no activity against human pathogens or marine bacteria [26,28,121,122].

Elatol from multiple *Laurencia* species (*L. chondrioides*, *L. majuscula*, and *L. obtusa*) mollusks (*A. dactylomela* and *A. parvula*) displayed either moderate or no activity against *E. coli* [26,35,36,72,99,117,118].

In addition, (−)-elatol, isolated from *L. dendroidea*, was also evaluated for its antimycobacterial potential, showing a moderate-to-high activity in the growth of *Mycobacterium bovis* BCG and not being able to inhibit the growth of *M. tuberculosis* [49].

These results indicate that the antibacterial and antimycobacterial activities of elatol are highly dependent on its source species and enantiomeric form, highlighting its potential as a bioactive lead compound while underscoring the need for the targeted evaluation for specific pathogens.

#### 5.5.2. Antifungal Activity

Elatol isolated from *L. obtusa* [72] and *L. rigida* [26] exhibited a moderate antifungal activity, inhibiting the growth of *Ustilago violacea*, *Fusarium oxysporum*, and human pathogen *Candida albicans*, while showing a strong activity against *Mycotypha microspora* and *Eurotium repens*. The ethanolic extract of *L. dendroidea*, containing elatol as identified through GC-MS analysis, also inhibited the growth of *Colletotrichum lagenarium*, although it was inactive against *Aspergillus flavus* [123]. Furthermore, Wessels et al. [35] demonstrated the antimicrobial properties of elatol isolated from *A. dactylomela* against *Microbotryum violacea* and *F. oxysporum* fungi strains.

Overall, these studies highlight that elatol exhibits selective antifungal activity, with an effectiveness dependent on both the fungal species and the source organism, underscoring its potential as a natural antifungal agent for further investigation.

#### 5.5.3. Antiviral Activity

Soares et al. [124] provided the first biological evidence of the antiviral potential of elatol. In a screening of 36 macroalgal extracts from the Brazilian coast, the extract of *Laurencia dendroidea*—in which (−)-elatol was the major constituent—displayed selective antiherpetic activity against acyclovir-resistant *Herpes simplex* viruses. The extract inhibited HSV-1 by 97.5% and HSV-2 by 43.8%, with a CC_50_ of 48.2 µg/mL.

More recently, in silico studies have expanded the range of viral targets associated with elatol, including those related to emerging pathogens such as SARS-CoV-2. Nag et al. [125] performed a molecular docking analysis of 24 phytochemicals against 4 essential viral proteins, identifying 11 compounds—including elatol—as promising candidates capable of interacting with RNA-dependent RNA polymerase (RdRp) and additional SARS-CoV-2 targets. In a subsequent virtual screening, Pokharkar et al. [126] evaluated elatol and other low-molecular-weight marine natural products as potential inhibitors of SARS-CoV-2 enzymes. Although elatol exhibited only weak binding scores, its interactions were structurally consistent with known pharmacophores for these proteins.

Taken together, these studies demonstrated that elatol possesses a measurable in vitro antiviral activity against herpes viruses and exhibits an in silico compatibility with key SARS-CoV-2 proteins. While experimental validation is still required for coronaviruses, the current evidence highlights elatol as a marine-derived metabolite with promising and potentially broad antiviral relevance.

Collectively, these results indicate that elatol is a marine-derived metabolite with a confirmed antiviral activity against herpes viruses and promising interactions with SARS-CoV-2 targets, highlighting the need for further experimental studies to explore its therapeutic potential.

### 5.6. Cellular Target

**Figure 7:**
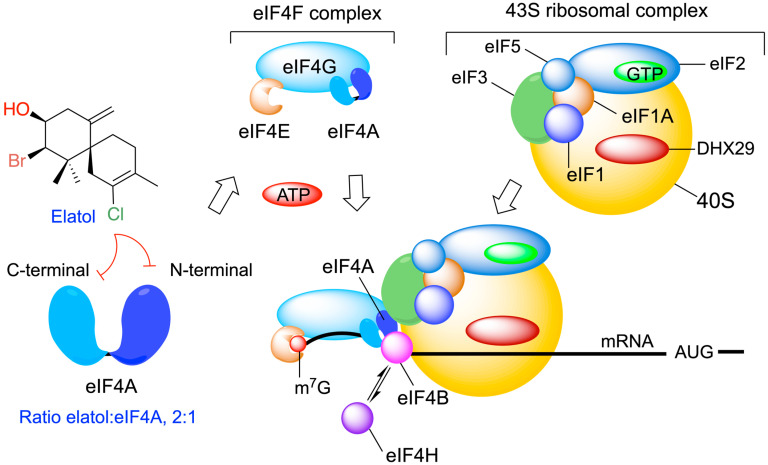
Cellular target for elatol on eIF4A (eukaryotic translation initiation factor 4A) and its key role in the translation of the mRNA. Elatol binds in a 2:1 ratio to eIF4A and disrupts helicase activity.

Elatol was proposed to be a specific inhibitor of ATP hydrolysis by eIF4A1 in vitro, exhibiting a broad activity against multiple tumor types. Molecular modeling studies predicted a 2:1 stoichiometry, suggesting the binding of two adjacent elatol molecules in the helicase core of eIF4A, with each elatol molecule interacting with a lysine residue from either the amino- or carboxy-terminal RecA-like domains of eIF4A (Figure 7) [127,128]. Moreover, these results highlight the ATP-binding pocket as a promising drug-binding site for the further development of eIF4A1 inhibitors.

Elatol’s identification as an eIF4A1 inhibitor with in vivo antitumor activities provides proof of principle for target-based screening against this highly promising target for cancer therapy.

### 5.7. Miscellaneous

The anti-inflammatory properties of (−)-elatol were demonstrated by Ventura et al. [49] through the evaluation of its antimycobacterial activity. The treatment of certain diseases, such as tuberculosis, involves the use of immunomodulatory compounds to inhibit pro-inflammatory mediator production by activated macrophages as a therapeutic strategy to prevent excessive inflammation. (−)-elatol significantly inhibited the production of NO and TNF-α in activated macrophages. This inhibitory effect on NO production was primarily mediated by the specific suppression of iNOS expression in activated cells. Then, (−)-elatol emerges as a selective anti-inflammatory agent by suppressing the macrophage production of key pro-inflammatory mediators.

Anticholinesterase compounds play a crucial role in the treatment of neurodegenerative disorders such as Alzheimer’s disease, which is characterized by a progressive cognitive decline associated with the degeneration of cholinergic neurons. These compounds act by inhibiting acetylcholinesterase (AChE), the enzyme responsible for degrading acetylcholine, thereby increasing its availability in the synaptic cleft. (−)-elatol has been associated with AChE inhibition, preventing acetylcholine breakdown and promoting its accumulation, which in turn enhances cholinergic signaling, which is essential for cognitive processes including learning and memory. Although the precise binding mode of (−)-elatol to AChE has not yet been fully elucidated, molecular docking analyses suggest that its bioactivity may result from interactions within the enzyme’s active site or regulatory regions, ultimately slowing the catalytic degradation of acetylcholine [54].

Elatol isolated from the marine mollusk *Aplysia dactylomela* has also been investigated for its antimutagenic properties [129]. In that study, crude extracts, fractions, and purified metabolites were assessed for their ability to inhibit 2-aminoanthracene (2AN)-induced mutagenicity using the *Salmonella typhimurium* TA98 strain in the Ames assay. The experimental design involved exposing the bacterial strain to a mixture containing the test compound, a metabolic activation system (S9 mix), and the standard mutagen 2AN. Among the bioactive metabolites, elatol markedly reduced the mutagenic response, demonstrating its capacity to mitigate 2AN-induced genetic damage. These findings provided some of the earliest evidence of the genoprotective potential of elatol and highlighted the compound as a promising lead for the development of antimutagenic agents.

## 6. Intellectual Property

The remarkable biotechnological potential of elatol has attracted growing academic and industrial interest due to its broad biological activities and eco-friendly profile. Patent searches revealed a limited yet increasing number of elatol-related inventions spanning agricultural, environmental, and biomedical applications. Here, we present the existing patents, integrated with sections on biosynthesis, ecology, biological activities, and ecological expressions, addressing the different potential uses involving elatol.

The earliest patent [130] described elatol-based formulations for controlling *Oncopeltus fasciatus* and *Trichoplusia ni*, insect pests responsible for extensive damage to cotton crops and Brassicaceae crops. This pioneering work demonstrated elatol’s efficacy across developmental stages while ensuring safety for crops and non-target organisms, establishing its potential as a sustainable, eco-friendly alternative to conventional synthetic insecticides and laying the foundation for further applications in agricultural biotechnology.

A series of Japanese patents [131,132,133,134,135,136,137,138] explored elatol’s ecological roles as an anti-herbivory agent. Increasing anthropogenic pressures, including marine pollution and ocean warming, have caused a sharp decline in marine farms cultivating commercially valuable seaweeds, as well as the associated loss of fauna and reduced fishery resources worldwide. In response, several technologies were developed to restore seaweed beds, including artificial reefs made from biodegradable materials and natural deterrents such as elatol, into ropes, nets, and controlled-release systems. These approaches aimed to protect seaweed farms while reducing environmental impact.

Later, Da Gama et al. [139] later claimed the use of elatol as a marine antifouling agent, proposing a sustainable alternative to toxic heavy metal-based coatings. Further developments combined elatol with other natural biocides, including capsaicin and cnidium lactone [140], to enhance antifouling performance while maintaining a low ecological impact. These multi-compound formulations improved the inhibition of fouling organisms and strengthened the potential of elatol as a key component of environmentally compatible antifouling technologies.

Beyond ecological uses, a significant body of patents addresses the biological and biomedical activity of elatol, primarily its anticancer potential. Elatol was identified as a specific inhibitor of ATP hydrolysis by eIF4A1, disrupting mRNA unwinding and translation initiation [127,128]. Subsequent patents exploited this mechanism in therapeutic strategies against leukemias, lymphomas, and prostate cancers, often in combination with other agents such as DHODH inhibitors to enhance selectivity, reducing the toxicity and overcoming resistance [141,142].

Patents by Hsieh (2021) proposed compositions targeting androgen receptor-deficient and castration-resistant prostate cancers through the inhibition of eIF4 complex enzymes [143,144,145]. In the same year, Hesson et al. [144] disclosed real-time dose-adjustment methods incorporating anticancer compounds into adaptive treatment protocols to optimize therapeutic efficacy and minimize adverse effects. Although elatol was cited as an embodiment, the scope of the claim did not cover the molecule itself but rather its application within the described method.

More recently, Bottley & Chapman [146] claimed eIF4A inhibitors, including elatol, for use in treating cancers, viral infections (e.g., influenza, Zika, SARS-CoV-2), CNS disorders, and inflammatory diseases. These claims also extended to nutraceutical and veterinary formulations (e.g., animal feed), positioning elatol not only as a direct therapeutic candidate but also as a molecular scaffold for the development of new translation-modulating agents. This breadth of applications reflects the growing interest in targeting the eIF4A axis across multiple disease classes and commercial sectors.

Hsieh & Jana [147] further disclosed methods for targeting SWI/SNF-mutant cancers, particularly ARID1A-deficient tumors, through the inhibition of eEF2-mediated translation elongation. This invention expands the mechanistic landscape in which elatol may operate, reinforcing translation control as a therapeutic vulnerability in genetically defined tumor subtypes and supporting its integration into precision oncology strategies.

Despite its promising in vitro and in vivo antiproliferative activity [148], elatol remains at the preclinical stage; however, its consistent modulation of translational control has guided the identification of new therapeutic targets. Indeed, recent patents, such as those targeting eIF4A1 and eEF2-mediated translation elongation, highlight elatol’s potential not only as a lead compound but also as a scaffold for next-generation translation-targeted therapies.

In addition to its investigation in oncology, elatol has attracted patent interest in other therapeutic areas, Fernández et al. [149,150] and Peixoto et al. [151,152] patented the use of (+)-elatol against the pathogenic amoeba *Naegleria fowleri*, the causative agent of primary amebic meningoencephalitis (PAM), broadening its pharmacological relevance beyond oncology. This innovation underscores elatol’s versatility as a bioactive natural product, extending its potential applications from cancer therapy to the treatment of highly lethal and neglected infectious diseases and highlighting the molecule as a promising candidate for future pharmacological development in areas of urgent medical need.

Despite this growing diversity of applied uses, the intellectual property surveyed remains largely oriented toward end-use applications, with limited attention to the biological origin and sustainable supply of the compound. None of the patents address elatol’s biosynthetic pathway, metabolic regulation, or scalable production strategies. This absence highlights a notable gap in the current patent domain and points to marine biotechnology, biosynthetic elucidation, and sustainable production platforms as underexplored yet strategically relevant avenues for future research and innovation.

Overall, the intellectual property landscape reveals that elatol has progressively moved from an ecologically relevant algal metabolite to a molecule of high strategic interest across multiple technological domains. Its capacity to modulate diverse biological processes, ranging from cellular fouling to translation control, has driven innovation both in sustainable environmental technologies and in advanced therapeutic strategies. Although commercial applications remain in their early stages, the breadth of patented uses demonstrates that elatol occupies a privileged position among marine natural products. This expanding trajectory strongly contributes to the emerging view of elatol as a potential *Smart Secondary Metabolite*, whose evolutionary and ecological functions have inadvertently positioned it as a molecule with broad biotechnological promise.

## 7. Trends, Challenges, and Future Perspectives

One of the striking features of many marine organisms is their enormous diversity of secondary metabolites, which poses a huge challenge: what drives the diversity of secondary metabolites in the organisms that produce them? *Laurencia* stands out as one of the richest sources of these chemicals [44], and, in this review, we reveal the success of one of many of these molecules, the sesquiterpene elatol, through its global occurrence, biosynthesis, broad ecological roles, and high biotechnological potential.

An interesting and comparative speculation concerning the huge profusion and diversity of the structures of natural products is that it represents an example of evolution in progress [153]. An important consequence of terpenoid pathway biosynthesis would provide one of the most versatile routes to compounds that are capable of transmitting information at all levels of biological organization [154]. Then, it provides a reservoir of non-functional variety out of which new functional processes can emerge in the future [154], or they are subsequently screened, as predicted by the screening hypothesis [155].

Elatol is a sesquiterpene isolated from only 13 of the 144 recognized *Laurencia* species, corresponding to roughly 8.5% of the genus. Despite this limited distribution, elatol plays a critical role in the ecological and biotechnological success of *Laurencia* given its widespread global occurrence and diverse adaptive function. The genus *Laurencia* is broadly distributed across tropical and temperate coastal regions, particularly in the Atlantic, Pacific, and Indian Oceans, reflecting its evolutionary success in diverse environmental contexts. The chemical diversity of *Laurencia*, especially its terpenoid metabolites, suggests that elatol is a key factor in the defense against herbivory, competitors, and fouling.

The biosynthesis of elatol and related terpenoids follows the classical mevalonate (MVA) and methylerythritol phosphate (MEP) pathways, generating a wide array of sesquiterpenes that provide adaptive advantages. The extensive secondary metabolite diversity in *Laurencia* raises an intriguing evolutionary question: what drives such chemical proliferation? One hypothesis posits that secondary metabolites first arise as chemical diversity “reservoirs,” which are then selectively retained for ecological expression [154]. In this view, elatol may have initially emerged as part of a chemically diverse repertoire and subsequently acquired adaptive ecological functions. This chemical diversity also promotes rapid evolutionary responses, as small structural variations in sesquiterpenes like elatol can lead to distinct ecological effects. The selective advantage conferred by elatol may thus reflect an example of *Smart Secondary Metabolite* evolution, where a compound originally selected for specific ecological roles also demonstrates a broad biotechnological relevance.

Elatol fulfills multiple ecological roles in *Laurencia*, functioning as an anti-herbivory, antifouling, and allelopathic agent. Remarkably, its spectrum of activity extends far beyond its ecological context, showing bioactivity against bacteria, fungi, viruses, protozoa, insect larvae, and cancer cells. This phenomenon illustrates a key aspect of *Smart Secondary Metabolites*: molecules selected for ecological functions can coincidentally exhibit bioactivities relevant for human applications. Marine natural products like elatol can modulate complex cellular processes, including apoptosis, angiogenesis, migration, and invasion, in both in vitro and in vivo models. The connection between ecological function and biomedical activity may be mediated by conserved cellular pathways, explaining why compounds selected through evolution for defense in marine environments may also serve as therapeutic leads.

A high intraspecific variation promotes the ability to evolve rapidly [155], and this seems to make sense if we consider *Laurencia*’s high chemical diversity and, above all, its various sesquiterpenes. In addition, the countless examples of the action of a single molecule, such as elatol, and the fact that small structural variations in other similar molecules result in different effects in ecological interactions reinforce the importance of chemical diversity. Then, this view makes sense when we look at *Laurencia* and its wealth of terpenoid metabolites; we might think that the right elatol would have emerged in various locations in the different oceans and today expresses itself with the most diverse ecological roles. The studies included in this review reveal how vital this adaptive chemistry represented by elatol is for the species that produce it, since it acts as a defense against natural adversities such as herbivory [74], competitors [69], and fouling [12].

Despite the promising biotechnological potential of elatol, several challenges remain, such as its sustainable production, since the scalable chemical or biotechnological synthesis is necessary to meet industrial demands while minimizing environmental impacts. Enantiomer-specific activity: Distinct enantiomers of elatol may differ in biological activity, requiring detailed stereochemical studies. Mechanistic elucidation: Although targets such as eIF4A1 and apoptosis pathways have been identified, many molecular mechanisms remain poorly understood. Linking adaptive chemistry to therapeutic relevance: The apparent disconnect between ecological and biotechnological functions offers a unique opportunity to explore evolution-informed drug discovery strategies.

As natural products have been optimized by natural selection to mediate ecological interactions rather than to serve as pharmacologically active agents, the proportion that exhibit significant biological activity upon screening is inevitably small [156]. Compounds from seaweeds are known to exhibit the ability to mediate specific inhibitory cellular processes, such as apoptosis, angiogenesis, migration, and invasion, in both in vitro and in vivo models [157]. Such observations support the connection between biologically active or therapeutic applications and their possible ecological roles. For example, evidence of the allelopathic action of marine sponge species against competitors has been suggested as a valuable subsidy for the prospecting of cytotoxic substances, since it is expressed through its action on cellular processes [158]. However, elatol represents an exemplary *Smart Secondary Metabolite*, combining adaptive ecological roles with unanticipated biotechnological utility. Understanding its biogeography, biosynthesis, ecological roles, and diverse bioactivities provides a model for exploring how natural products can transcend their evolutionary origins to serve as innovative tools for medicine, biotechnology, and sustainable applications.

## 8. Conclusions

The specialized chemical machinery of certain marine organisms such as *Laurencia* spp. has yielded a series of natural compounds with unique characteristics, which are here defined for the first time as *Smart Secondary Metabolites*. These privileged classes of natural compounds are characterized by the simultaneous combination of several key features. They possess a singular chemical structure, and they are produced in notably high amounts. These metabolites may serve as biogenetic precursors to structurally related compounds. Additionally, their chemical structure confers to them a high versatility to interact with multiple biological targets, whether through promiscuous or highly specific binding interactions, so they exhibit potent biological activities across diverse systems. Thus, elatol belongs to the chamigrane class of halogenated sesquiterpenes, with a singular chemical structure characterized by a spiro [5.5] undecane skeleton containing highly functionalized rings with three stereocenters. Its key features include a methylidene group, a bromine atom, a hydroxyl group, and a methyl and a chlorine atom in an intramolecular double bond. Highly predominant and abundant in *Laurencia* species, elatol shows extremely high intra- and inter-populational variability. Elatol plays a multifunctional ecological role by mediating competitive interactions as an allelopathic agent, deterring consumers and pathogens, preventing fouling, and acting as a chemical cue in interactions with specialist herbivores. Elatol has demonstrated broad pharmacological potential, including anticancer, antimicrobial, antiparasitic, anti-cholinesterase, acaricidal, and insecticidal activities. Elatol has also been proposed as an eIF4A1 inhibitor, supporting its potential as a target-based cancer therapy. Its wide range of biological activities and eco-friendly profile have attracted growing academic and industrial interest, reflected in an increasing number of patents in agriculture, environmental, and biomedical applications. In summary, elatol is a singular, multifunctional metabolite whose distinctive chemical structure, ecological roles, and diverse biotechnological and pharmacological activities make it a highly promising *Smart Secondary Metabolite*.

## Data Availability

No new data were created or analyzed in this study. Data sharing is not applicable to this article.

## Supplementary Materials

The following supporting information can be downloaded at https://www.mdpi.com/article/10.3390/md24020061/s1, Figure S1: Flow diagram of study selection following PRISMA 2020 guidelines. Records were identified through database searches, duplicates were removed, titles and abstracts were screened, full texts were assessed for eligibility based on predefined criteria, and only studies directly addressing the naturally occurring compound elatol were included; Table S1: Collection sites/oceans, pharmacological activities, and ecological roles of elatol isolated from *Laurencia* species; Table S2: Collection sites/oceans, pharmacological activities, and ecological roles of elatol isolated from marine invertebrates [12,16,17,18,20,21,22,23,24,25,26,27,28,29,30,31,32,33,34,35,36,37,38,43,47,48,49,50,51,52,53,54,63,65,66,67,68,69,70,71,72,73,74,75,78,87,92,93,94,95,97,98,99,100,101,102,103,104,106,108,112,113,117,118,119,120,121,122,123,124,125,126,127,129,159,160,161,162,163].
