# Supplementary material for: Smart Secondary Metabolites in Marine Environments: The Case of Elatol
Source: Mar Drugs. 2026 Feb 1;24(2):61. doi: 10.3390/md24020061 (PMC12942527; doi:10.3390/md24020061)
Supplement: Supplementary file 1 [file marinedrugs-24-00061-s001.zip › marinedrugs-4067143-supplementary.pdf]

Supplementary Materials

List of Contents

Methodological Search Framework **S2**

Figure S1 Flow diagram of study selection following PRISMA 2020 guidelines. Records were identified through database searches, duplicates were removed, titles and abstracts were screened, full texts were assessed for eligibility based on predefined criteria, and only studies directly addressing the naturally occurring compound elatol were include **S2**

Table S1 Collection sites/Oceans, pharmacological activities and ecological roles of elatol isolated from *Laurencia* species **S5**

Table S2 Collection sites/Oceans, pharmacological activities and ecological roles of elatol isolated from marine invertebrates **S23**

References **S26**

Methodological Search Framework

To ensure methodological transparency, the study selection process followed the PRISMA 2020 framework (identification, screening, eligibility, and inclusion), ensuring high specificity and conceptual coherence in the literature focused on elatol. A simplified flow diagram reporting the number of records at each stage is provided in Figure S1.

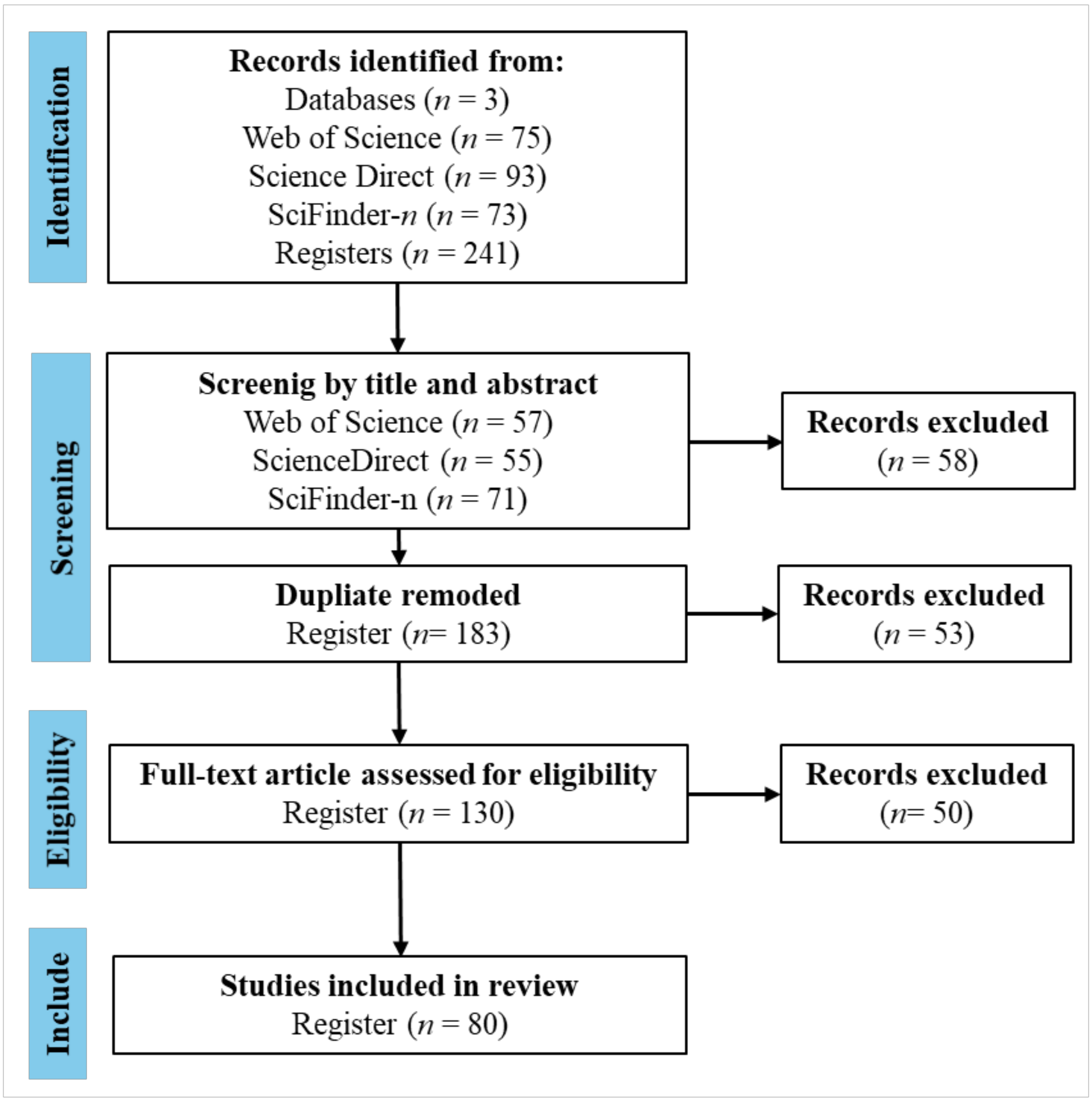

Figure S1. Flow diagram of study selection following PRISMA 2020 guidelines. Records were identified through database searches, duplicates were removed, titles and abstracts were screened, full texts were assessed for eligibility based on predefined criteria, and only studies directly addressing the naturally occurring compound elatol were included.

Below is a detailed description of the procedures used in the search and workflow for selecting relevant articles:

1. Search Strategy and Data Sources

Records were identified through a systematic search of Web of Science, ScienceDirect, and SciFinder-n covering publications from 1974 to 2025. Searches were last updated in November

2025. The search was based on the keyword “elatol”, applied across all searchable fields. The IUPAC official nomenclature was not used for searching. No Boolean operators or truncations were used, to maintain a highly specific strategy focused on the target compound. Only peer-reviewed journal articles published in English were considered. Other document types, including conference abstracts, books, book chapters, editorials, and technical reports, were excluded. Patents were not included, as they were analysed separately in another section of the manuscript.

2. Study Selection and Screening Procedure

The selection process followed a structured, multistep workflow:

2.1. Title and Abstract Screening: An initial screening based on titles and abstracts was performed to identify studies addressing elatol and to remove non-research formats and clearly irrelevant records.

2.2. Deduplication: Duplicate records across databases were identified and removed using Digital Object Identifiers (DOI) when available. When DOI information was missing, duplicates were detected by exact matching of article titles and author names.

2.3. Full-Text Assessment: Full texts of potentially eligible articles were examined to determine compliance with predefined inclusion and exclusion criteria. Articles in which elatol was not the primary subject were excluded at this stage.

3. Eligibility Criteria

3.1. Inclusion Criteria

Studies were included if they met explicitly at least one of the following conditions:

(i) Investigated elatol in ecological contexts, including chemical defense, species interactions, or environmental roles.

(ii) Evaluated the pharmacological or biological activities of elatol, such as antimicrobial, antiparasitic, cytotoxic, or other bioactivities of elatol from natural sources.

(iii) Reported the isolation and chemical characterization of elatol from natural sources, providing analytical or structural data.

Only information explicitly reported in the original publications was collected.

3.2. Exclusion Criteria

Studies were excluded when they:

(i) Focused on chemical synthesis, semi-synthesis, structural analogues, biotransformations, or derivative compounds of elatol.

(ii) Mentioned elatol only marginally, without presenting empirical, ecological, biological, or analytical data.

(iii) Corresponded to non-peer-reviewed formats (conference proceedings, editorials, commentaries, opinion pieces, or books/book chapters lacking methodological support).

4. Data Collection Process

From each eligible article, data were extracted and compiled into two standardized tables (Tables S1 and S2). Extracted variables included: Stereochemistry (when available), Alga or invertebrate species as natural source, Collection sites, Biological and ecological activity. Only information explicitly reported in the original publications was collected.

Table S1. Collection sites/Oceans, pharmacological activities and ecological roles of elatol isolated from *Laurencia* species.

Compound	Alga Specie	Collection Site (Latitude)	Ocean	Approach	Biological Activity							Reference
						Pharmacological						
					Ecological	Antiproliferative	*In vivo* Antitumoral	Antiparasitic	Insecticidal and Acaricidal	Antimicrobial	Miscel	
[†]elatol	*Laurencia cartilaginea*	Ma'ili Pt. Park, Wai'anae coast of O'ahu, Hawai, USA (21º N)	Pacific	Pharmac		IC$_{50}$ (µM): P-388 (3.0), A-549 (0.3), HT-29 (0.3) and MEL-28 (0.3)						Juagdan *et al.* 1997
[†]elatol	*L. chondrioides*	Gran Canaria, Canary Islands, Spain (28º N)	Atlantic	Pharmac	Antibacterial (200 µg/disc), inhibition zones (mm): *Vibrio anguillarum* (3.0), *Pseudomonas anguilliseptica* (4.0)					Antibacterial (200 µg/disc), inhibition zones (mm): *Staphylococcus aureus* (1.0)		Bansemir *et al.* 2004
[†]elatol	*L. decumbens*	Weizhou Island, China (21º N)	Pacific	Chemical[#]								Ji *et al.* 2007a
[†]elatol	*L. dendroidea***	Cabo Frio Island, Rio de Janeiro (RJ) State, Brazil (22º S)	Atlantic	Ecological	Antifouling: mussel *Perna perna*							Da Gama *et al.* 2003

†elatol	*L. dendroidea***	Cabo Frio Island, (RJ) State, Brazil (22º S)	Atlantic	Ecological	Antiherbivory: crab *Pachygrapsus transversus* and the sea urchin *Lytechinus variegatus.* Antifouling: field assays		Pereira *et al.* 2003
†elatol*	*L. dendroidea***	Cabo Frio Island (RJ) State, Brazil (22º S)	Atlantic	Ecological	Surface and intrathalli. Quantification by GC-ECD		Sudatti *et al.* 2006
†elatol	*L. dendroidea***	Cabo Frio Island, RJ State, Brazil (22º S)	Atlantic	Ecological	Intra- and Intercellular transport Antifouling: barnacle larvae *Amphibalanus* sp. Antiherbivory: sea urchin *Lytechinus variegatus*)		Sudatti *et al.* 2008
†elatol	*L. dendroidea*	Cabo Frio Island, RJ State, Brazil (22º S)	Atlantic	Pharmac		IC_{50} **(**µM) Antileishmanial *Leishmania amazonensis*: promastigote (4.0 ± 0.3) and amastigote (0.45)	Dos Santos *et al.* 2010

†elatol *	*L. dendroidea***	Cabo Frio Island, RJ State, Brazil (22º S)	Atlantic	Ecological	Stress-triggered exocytosis from *corps en cerise* to algal surface			Paradas *et al.* 2010
†elatol	*L. dendroidea*	Cabo Frio Island, RJ State, Brazil (22º S)	Atlantic	Pharmac		IC_{50} (µM) Antichagasic *Trypanosoma cruzi*: promastigote (45.4 ± 1.9), trypomastigote (1.38 ± 0.15) and amastigote (1.01 ± 0.65)		Veiga-Santos *et al.* 2010
†elatol *	*L. dendroidea*	Cabo Frio Island, RJ State, Brazil (22º S)	Atlantic	Ecological	Physical and nutritional effects on elatol levels. Quantification by GC-ECD			Sudatti *et al.* 2011
(-)-elatol	*L. dendroidea*	Cabo Frio Island, RJ State, Brazil (22º S)	Atlantic	Pharmac		Antichagasic *Trypanosoma cruzi*: trypomastigote (80.0% reduction of mitochondrial depolarization, 3.0 µM)		Desoti *et al.* 2012
(-)-elatol*	*L. dendroidea*	Cabo Frio Island, RJ State, Brazil (22º S)	Atlantic	Pharmac			Antiviral *Herpes simplex*: HSV-1-ACVr (97.5 %), HSV-2-ACVr (43.8 %)	Soares *et al.* 2012

(-)-elatol	*L. dendroidea*	Cabo Frio Island, RJ State, Brazil (22º S)	Atlantic	Pharmac		Antichagasic *Trypanosoma cruzi*: amastigote (60.0% reduction of mitochondrial depolarization, 3.0 µM)	Desoti *et al.* 2014
†elatol *	*L. dendroidea*	Cabo Frio Island, RJ State, Brazil (22º S)	Atlantic	Ecological	Diel variation. Quantification by GC-ECD		Sudatti *et al.* 2016
†elatol*	*L. dendroidea*	Cabo Frio Island, RJ State, Brazil (22º S)	Atlantic	Ecological	Antifouling (microalga and bacterium) induced by herbivory		Sudatti *et al.* 2018
(-)-elatol	*L. dendroidea*	Biscaia inlet, Angra dos Reis, RJ State, Brazil (23º S)	Atlantic	Pharmac		IC_{50} **(**µM) Antileishmanial *Leishmania amazonensis*: promastigotes (29.1 ± 3.6) and amastigotes (13.5 ± 2.7)	Machado *et al.* 2011
(-)-elatol*	*L. dendroidea*	Biscaia inlet, Angra dos Reis, RJ State, Brazil (23º S)	Atlantic	Pharmac		IC_{50} **(**µM) Antileishmanial *Leishmania amazonensis*: promastigotes (53.6 ± 3.9) and	Machado *et al.* 2014

						amastigotes (67.1 ± 9.6)			
(-)-elatol	*L. dendroidea*	Biscaia inlet, Angra dos Reis, Rio de Janeiro State, Brazil (23º S)	Atlantic	Pharmac			Antimycobacterial: *Mycobacterium bovis Bacillus* Calmette–Guérin and *M. tuberculosis* $H_{37}Rv$)	IC_{50} (µM): Nitric oxide (49.5 ± 3.2) and TNF-α production (189.8 ± 3.6)	Biá Ventura *et al.* 2015
(-)-elatol	*L. dendroidea*	Biscaia inlet, Angra dos Reis, RJ State, Brazil (23º S)	Atlantic	Pharmac	IC_{50} **(**µM): Colo-205 (7.58 ± 4.13), B16F10 (54.65 ± 4.74), Jurkat (31.31 ± 3.41) and U937 (37.34 ± 3.62)				Barcellos Marini *et al.* 2018
(-)-elatol<br><br>(+)-elatol	*L. dendroidea*	Biscaia inlet, Angra dos Reis, RJ State, Brazil (23º S)<br><br>Azeda beach, Armação de Búzios, RJ State, Brazil (22º S)	Atlantic	Pharmac				AchE activity (% at 300 µM): (78.5)<br><br>AchE activity (% at 300 µM): not active	Gonçalves *et al.* 2020
(-)-elatol<br><br>(+)-elatol	*L. dendroidea*	Biscaia inlet, Angra dos Reis, RJ State, Brazil (23º S)<br><br>Azeda beach, Armação de Búzios, RJ	Atlantic	Pharmac		IC_{50} **(**µM) (Trophozoites of *Naegleria fowleri*): ATCC 30808 (36.77 ± 8.03) and ATCC 30215 (38.03 ± 7.61)			Arberas-Jiménez *et al.* 2023

		State, Brazil<br>(22º S)				ATCC 30808<br>(1.08 ± 0.09)<br>and ATCC<br>30215 (1.14 ±<br>0.09)		
(-)-elatol	*L. dendroidea*	Macaé, RJ<br>State, Brazil<br>(22º S)	Atlantic	Pharmac		IC_{50} (μM)<br>Antileishmanial:<br>*L. amazonensis*<br>promastigote:<br>121.7 ± 4.8		Machado *et al.* 2014
†elatol *	*L. dendroidea*	Azeda beach,<br>Armação de<br>Búzios, RJ<br>State, Brazil<br>(22º S)<br><br>Biscaia inlet,<br>Angra dos<br>Reis, RJ State,<br>Brazil<br>(23º S)<br><br>Vermelha<br>Beach, Paraty,<br>RJ State,<br>Brazil<br>(23º S)	Atlantic	Ecological	Geographic<br>quantitative<br>variation.<br>Quantification<br>by GC-MS			Machado *et al.* 2016
(-)-elatol	*L. dendroidea*	Vermelha<br>Beach, Paraty,<br>RJ State,<br>Brazil<br>(23º S)	Atlantic	Pharmac			Larvicidal<br>(% *Aedes*<br>*aegypti* at<br>10 ppm):<br>(30%)	Salvador-Neto *et al.* 2016
(-)-elatol	*L. dendroidea*	Ubu and<br>Castelhanos<br>beach,<br>Anchieta,<br>Espírito Santo	Atlantic	Pharmac		LD_{100} (μM)<br>Antischistosom<br>al *Schistosoma*<br>*mansoni*: adult<br>worms (149.8)		Santos *et al.* 2022

		State, Brazil<br>(20º S)<br><br>Brava beach, Ubatuba, São Paulo State, Brazil<br>(23º S)							and cercariae (37.5). *Biomphalaria glabrata,* schistosomose vector: blastulae (4.67) and veliger (18.7)				
(+)-elatol	*L. dendroidea*	Azeda beach, Armação de Búzios, RJ State, Brazil (22º S)	Atlantic	Ecological	Attracting cues for specialist herbivore *Aplysia brasiliana*								Nocchi *et al.* 2017
(+)-elatol	*L. dendroidea*	Azeda beach, Armação de Búzios, RJ State, Brazil (22º S)	Atlantic	Ecological	Anti-herbivory defenses induced by conspecific cues								Pereira *et al.* 2020
†elatol*	*L. dendroidea***	Rasa Beach, Armação de Búzios, RJ State, Brazil (22º S)	Atlantic	Ecological	Exocytosis, storage and characterization of corps *en cerise*								Salgado *et al.* 2008
†elatol*	*L. dendroidea*	Armação de Búzios, RJ State, Brazil (22º S)	Atlantic	Pharmac							Antifungal by bioatuotgraphy: *Colletotrichum lagenarium*		Fernandes Peres *et al.* 2012
†elatol	*L. dendroidea*	Boa Viagem Beach, Recife, Pernambuco State, Brazil (8º S)	Atlantic	Pharmac						Acaricidal (% mortality *Tetranychus urticae at*			Born *et al.* 2012

						2.6μg/cm^2): (40.0 ± 1.6%)		
†elatol	*L. dendroidea*	Suape Beach, Pernambuco State, Brazil (8º S)	Atlantic	Pharmac		Larvicidal (LC_{50} *Aedes aegypti*): (10.7 ± 0.4 ppm)		Bianco *et al.* 2013
†elatol	*L. dendroidea*	Suape Beach, Pernambuco State, Brazil (8º S)	Atlantic	Pharmac			Antibacterial MIC (μg/mL): *Mycoplasma hominis* (ATCC23114), *M. genitalium* (ATCC33530), *M. capricolum* (ATCC27343), *M. pneumoniae* strain 129 (ATCC29342) and FH (ATCC15531): (all >100)	Bianco, *et al.* 2015
†elatol*	*L. dendroidea*	Vilas do Atlântico Beach, Lauro de Freitas, Bahia State, Brazil (12º S)<br>Castelhanos Beach, Anchieta, Espírito Santo State, Brazil (20º S)	Atlantic	Ecological	Inter- and intrapopulation quantitative variation. Quantification by GC-ECD			Oliveira *et al.* 2013

		Forno Inlet, Armação dos Búzios, RJ State, Brazil (22º S)<br><br>Velho Beach, Angra dos Reis, Rio de Janeiro state, Brazil (23º S)				
†elatol*	*L. dendroidea*	Vilas do Atlântico Beach, Lauro de Freitas, Bahia State, Brazil (12º S)<br><br>Castelhanos Beach, Anchieta, Espírito Santo State, Brazil (20º S)<br><br>Forno Inlet, Armação dos Búzios, RJ State, Brazil (22º S)<br><br>Velho Beach, Angra dos Reis, RJ State, Brazil (23º S)	Atlantic	Ecological	Production variability under common garden conditions x field individuals. Quantification by GC-ECD	Sudatti *et al.* 2021
†elatol*	*L. dendroidea*		Atlantic	Ecological	Allelopathy and autotoxicity	Sudatti *et al.* 2020

		Forno Beach, Armação dos Búzios, RJ State, Brazil (22º S)<br><br>Azeda Beach, Armação dos Búzios, RJ Sstate, Brazil (22º S)											
†elatol*	*L. dendroidea*	Rasa Beach, Búzios, RJ State, Brazil (22º S)	Atlantic	Ecological	Biochemical and cellular approaches to ABC transporter localization								Salgado *et al.* 2023
(+)-elatol	*L. elata*	Coast of New South Wales, Australia (33-37º S)	Pacific	Chemical# (First isolation)									Sims & Wing 1974
(+)-elatol	*L. majuscula*	Woodmans Point, Perth, Australia (32º S)	Indian	Chemical#									Capon *et al.* 1988
(+)-elatol	*L. majuscula*	Pulau Tikus, Sandakan, Sabah, Malaysia (6º N)<br><br>Pulau Nunuyan Laut, Sandakan, Sabah, Malaysia (6º N)	Indic	Pharmac	Geographic variation<br><br>Antibacterial (90 µg/disc) Inhibition zones (mm): *Clostridium novyi* (7–12), *C. sordellii* (7–12), *C. cellobioparum* (25–30), *Flavobacterium*						Antibacterial (90 µg/disc) Inhibition zones (mm): *Chromobacterium violaceum* (12–18), *Clostridium fallax* (7–12), *Escherichia coli* (7–12), *Enterobacter aerogenes* (7–12), *Shigella flexneri*		Vairappan *et al.* 2001

		Pulau Redang,<br>Terengganu,<br>Malaysia<br>(6º N)			*helmiphilum*<br>(19–24), *V.*<br>*vulnificus* (7–12)	(7–12), *Proteus*<br>*mirabilis* (25–30),<br>*Vibrio cholerae*, *V.*<br>*parahaemolyticus*<br>(7–12)	
(+)-elatol	*L. majuscula*	Pulau<br>Bankawan,<br>Sandakan,<br>Sabah,<br>Malaysia<br>(6º N)	Indic	Pharmac		Antibacterial<br>(90 µg/disc)<br>Inhibition zones<br>(mm):<br>*Chromobacterium*<br>*violaceum* (12–<br>18), *Clostridium*<br>*fallax* (7–12),<br>*Escherichia coli*<br>(7–12),<br>*Enterobacter*<br>*aerogenes* (7–12),<br>*Shigella flexneri*<br>(7–12), *Proteus*<br>*mirabilis* (25–30),<br>*Vibrio cholerae*, *V.*<br>*parahaemolyticus*<br>(7–12)	Vairappan,<br>*et al.* 2003
†elatol	*L. majuscula*	Gran Canaria,<br>Canary<br>Islands, Spain<br>(27º N)	Atlantic	Chemical			Masuda *et*<br>*al.* 1998
†elatol	*L. majuscula*	Spratly<br>Islands,<br>Malaysia<br>(8º N)	Pacific	Ecological<br>Pharmac	Antibacterial:<br>*Clostridium*<br>spp., *Proteus*<br>*vulgaris*, *Vibrio*<br>spp.	Antibacterial:<br>*Bacillus cereus*,<br>*Enterococcus*<br>*faecalis*,<br>*Escherichia coli*,<br>*Proteus mirabilis*,<br>*Pseudomonas*<br>*aeurelis*,<br>*Staphylococcus*<br>*aureus*, *Listeria*	Vairappan &<br>Siew-Moi<br>2005

						monocytogenes, *Salmonella* sp.<br><br>Antifugal: *Candida albicans*, *Cryptococcus neoformans*	
†elatol	*L. majuscula*	La Gomera, Canary Islands, Spain (28º N)	Atlantic	Chemical			Díaz-Marrero *et al.* 2009
†elatol	*L. majuscula*	Semporna district, Malaysia (4º N)	Pacific	Ecological Pharmac	Antibacterial (30 µg/disc). Inhibition zones (mm): *Alteromonas* sp1 (25-30), *Alteromonas* sp2 (12-18), *Proteus mirabilis* (12-18), *Proteus* sp. (7-12), *Cytophaga–Flavobacterium* (25-30), *Vibrio* sp. (25-30)	Antibacterial (30 µg/disc). Inhibition zones (mm): *Staphylococcus aureus* (7-12), *Staphylococcus* sp. (7-12), *Streptococcus* sp. (0), *Citrobacter freundii* (0), *Escherichia coli* (0), *Klebsiella pneumoniae* (0)	Vairappan, *et al.* 2010
†elatol*	*L. majuscula*	Borneo Island, Sabah,	Pacific	Ecological	Sequestration and bioaccumulatio		Palaniveloo & Vairappan 2014

		Malaysia (4º N)			n by the sea hare *Aplysia* sp.				
†elatol	*L. majuscula*	Selalong Island, Kota Kinabalu, Sabah, Malaysian (5º N)	Pacific	Pharmac				Antibacterial MIC-MBC (µg/ml): *Escherichia coli* (100-250), *Salmonella typhi* (100-250), *Vibrio cholera* (100-250)	Kamada *et al.* 2017
(+)-elatol	*L. majuscula*	Mantanani Island Borneo, Sabah, Malaysian (4º N)	Pacific	Pharmac		IC_{50} (µM): HeLa (2.25), MCF-7 (2.25) and P-388 (3.0)			Kamada *et al.* 2019
†elatol	*L. mariannensis*	Hainan and Weizhou Islands, China (21º N)	Indic	Chemical					Ji *et al.* 2007b
†elatol	*L. microcladia*	Sepultura Beach, Santa Catarina State, Brazil (27º S)	Atlantic	Pharmac	Antiherbivory: sea urchin *Echinometra lucunter*				Lhullier *et al.* 2009
†elatol	*L. microcladia*	Sepultura Beach, Santa Catarina state, Brazil (27º S)	Atlantic	Pharmac		IC_{50} (µM): L929 (1.1) > DU145 > MCF7 > A549 > B16F10 (10.1)	B16F10 in C57BL6 mice, mg/kg, tumor reduction (%) p.o: (30-61.2%), i.p: (10-71.4%)		Campos *et al.* 2012

†elatol	*L. microcladia*	Sepultura Beach, Santa Catarina State, Brazil (27º S)	Atlantic	Pharmac		CC_{50} (μM): A549 (7.56 ± 0.19), RD (11.22 ± 1.63)		Lang *et al.* 2012
†elatol	*L. microcladia*	No date	-	Pharmac		CC_{50} (μM): CML: (1.0), DLBCL: (1.0)		Maiti et al., 2021, Cunningham et al. 2022
(+)-elatol	*L. obtusa*	Canary Islands, Spain (28º N)	Atlantic	Chemical				González *et al.* 1976
†elatol	*L. obtusa*	Carrie Bow Cay, Belize (16º N)	Atlantic	Ecological Pharmac	Sequestration and bioacumulation from *Laurencia obtusa* by the sea hare *Aplysia dactylomella*	Total inhibition of sea urchin (*Strongylocentrotus purpuratus*) egg development at 16 g/mL	Antibacterial: *Staphylococcus aureus, Bacillus subtilis, Escherichia coli, E. castellani, E. chalmers and Candida albicans* Antifungal: *Candida albicans*	Norris & Fenical, 1982
(+)-elatol	*L. obtusa*	Discovery Bay, Jamaica (18º N)	Atlantic	Ecological Pharmac	Toxicity to brine shrimp (78 % mortality)		Antifungal: *Cladosporium cucuwinum*	Brennan *et al.* 1987

(+)-elatol	*L. obtusa*	Isleta Marina, Puerto Rico (18º N)	Atlantic	Chemical		Gerwick & Lopez 1987
†elatol	*L. obtusa*	No date	-	Ecological	Antiherbivory: sea urchin *Diadema antillarum* and reef fishes	Hay *et al*. 1987
†elatol	*L. obtusa*	Negril, Jamaica (18º N)	Atlantic	Chemical		Kennedy *et al.* 1988
†elatol	*L. obtusa*	Gran Canaria Canary Islands, Spain (27º N)	Atlantic	Chemical		Martin *et al.* 1989
†elatol	*L. obtusa*	Lanzarote, Canary Islands, Spain (27º N)	Atlantic	Chemical		Martin *et al.* 1989
†elatol	*L. obtusa*	Dos Roques beach, Gran Canaria, Canary Islands, Spain (28º N)	Atlantic	Ecological	Antiherbivory: gastropods *Osilinus atratus* and *Littorina striata*. Fish larva toxicity: gilthead bream *Sparus aurata*	Granado *et al.* 1995
†elatol *	*L. pacifica*	Playa Estación, Golf of California, México (23-37º N)	Pacific	Ecological	Chemotaxonomy by thyn layer cromatography	Fenical & Norris 1975
†elatol	*L. rigida*		Pacific	Ecological		De Nys *et al.* 1996

		Cape Banks, New South Wales, Australia (34º N)			Antifouling (barnacle *Balanus amphitrite* and bryozoan *Bugula neritina* larvaes). No strong inhibition of bacterial growth (*Vibrio fischeri* and *Serratia* sp.). Low activity against settlement and germination of the alga *Ulva lactuca*		
†elatol	*L. rigida*	Cape Banks, New South Wales, Australia (34º N)	Pacific	Pharmac		Inhibition zones (mm) Antibacterial: *Bacillus magaterium* (2), *Escherichia coli* (0) Antifungi: *Ustilago violacea* (3), *Mycotypha microspora* (25), *Eurotium repens* (25), *Fusarium oxysporum* (7) Antimicroalgal: *Chlorella fusca* (2)	König & Wright 1997
†elatol	*L. scoparia*	Negril, Jamaica (18º N)	Atlantic	Chemical#			Kennedy *et al.* 1988

†elatol	*L. similis*	Hainan and Weizhou, China (21º N)	Pacific	Chemical				Ji *et al.* 2007c
†elatol	*Laurencia* spp.	Guerilla Bay, New South Wales, Australia (35º S)	Pacific	Chemical				Rose & Sims 1977
(+)-elatol	*Laurencia* spp.	No date	-	Pharmac		% Adherence at 24 h **(**µM) Anti-*Naegleria* (Trophozoites of *N. fowleri*): ~0.10% (1.50), ~0.02% (3.00)		Chao-Pellicer *et al.* 2025
(+)-elatol	No date	No date	-	Pharmac	IC_{50} **(**µM): eIF4A1 inhibitor (16.4)			Peters *et al.* 2018
†elatol	No date	No date	-	Pharmac			*In silico* activity against SARS-CoV 2. Swiss ADME: TPSA: 20.23, iLOGP: 3.22, ESOL LogS: -4.52, ESOL class: Moderately Soluble, Lipinski#violations : 1, Leadlikeness#violations: 1, Bioavailability Score: 0.55 Proteins affinity (Kcal/mol):	Nag *et al.* 2021

						5WRG: -7.405, 65W4B: -7.58, 6Y84: -7.124, 6M71: -6.921	
†elatol	No date	No date	-	Pharmac	CC_{50} (μM): CML: (1.0), DLBCL: (1.0)		Cunningham *et al.* 2022
†elatol	No date	No date	-			*In silico* activity against SARS-CoV-2(Kcal/mol): RdRp protein (-5 to -5.9), nsp15 enzyme (-5 to -5.9)	Pokharkar *et al.* 2023

Pharmac= Pharmacological. #Chemical data available. †Stereochemistry not reported. * Identified in the extract; ** as *L. obtusa*. Miscel = Miscellaneous. LD= Lethal dose. IC= Inhibitory Concentration. CC= Cytotoxic Concentration. MIC= Minimum Inhibitory Concentration.

P-388: murine lymphocytic leukemia; MCF-7: human breast carcinoma; HeLa: human cervical epithelioid carcinoma; A-549: human lung carcinoma; HT-29: female colorectal adenocarcinoma; MEL-28: skin malignant melanoma; Colo-205: mixed adherent/suspension epithelial cell line; Jurkat: human T lymphocyte; U937: pleural histiocytic lymphoma; RD: human embryo rhabdomyosarcoma; L929: murine fibroblast; B16F10: murine melanoma; DU145: human prostate carcinoma, MCF-7: human mammary adeno-carcinoma. CML: Chronic myelogenous leukemia patient cells. DLBCL: diffuse large B-cell lymphoma. p.o.: Oral route of administration. i.p.: Intraperitoneal route of administration

Table S2. Collection sites/Oceans, pharmacological activities and ecological roles of elatol isolated from marine invertebrates.

Coupound	Invertebrate Specie	Collection Site (Latitude)	Ocean	Approach	Biological Activity						Reference
					Ecological	Pharmacological					
						Antiproliferative	Toxicity	Antiparasitic	Antimicrobial	Miscel	
(+)-elatol	*Aplysia dactylomela*	La Parguera, Puerto Rico (17º N)	Atlantic	Ecological	Sequestration and bioaccumulation from *Laurencia obtusa*. First isolation from the sea hare *Aplysia dactylomella*						Schmitz *et al.* 1982
(+)-elatol	*A. dactylomela*	San Juan de la Rambla, Tenerife, Canary Islands, Spain (28º N)	Atlantic	Pharmac		IC_{50} (µM): HM02 (<3.0), HEP G2 (<3.0), MCF 7 (<3.0)	*Artemia salina* LD_{100} (0.5 mg/mL)	IC_{50} (µM), MIC (µg/mL) Antichagasic (*Trypanosoma cruzi*): (2.76, 3.3) Nematicidal: *Caenorrhabditis elegans*	Antimicrobial (50 µg/disc). Inhibition zones (mm): Bacteria: *Bacillus megaterium* (2) Fungi: *Fusarium oxysporum* (7.0), *Microbotryum violacea* (3.0) Alga: *Chlorella fusca* (0)		Wessels *et al.* 2000
†elatol	*A. dactylomela*	La Palma, Canary Islands, Spain (28º N)	Atlantic	Pharmac		IC_{50} (µM): HeLa (12.3, 3.99), Hep-2 (7.2, 6.0), Vero (6.9, 133.6)					Dias *et al.* 2005
(+)-elatol	*A. dactylomela*	Salinas Bay, Cabo Rojo, Puerto Rico (17º N)	Atlantic	Chemical#							Vera *et al.* 2009

†elatol	*A. dactylomela*	La Gomera, Canary Islands, Spain (28º N)	Atlantic	Chemical$^{\#}$			MIC (μg/mL). Bacteria *Bacillus cereus* (> 50)		Díaz-Marrero *et al.* 2012
†elatol	*A. dactylomela*	No date	-	Pharmac				Mutagenicity on *Salmonella typhimurium*)	Wall 1992
†elatol	*Aplysia dactylomela*	Mona Island, Puerto Rico (18º N)	Atlantic	Chemical					Jiménez-Romero *et al.* 2014
†elatol	*A. dactylomela*	Sulug Island, Kota Kinabalu, Sabah, Malaysia (5º N)<br><br>Dinawan Island, Kota Kinabalu, Sabah, Malaysia (5º N)	Indic	Ecological	Sequestration and bioaccumulation from *Laurencia* sp.				Palaniveloo & Vairappan 2014
(+)-elatol	*A. parvula*	Sepanggar Island, Kota Kinabalu, Sabah, Malaysia (6º N)	Indic	Ecological Pharmac	Sequestration and bioaccumulation from *Laurencia* sp. Antibacterial: no date	IC$_{50}$ (μM): P388 (74.9)	Antibacterial: no date		Vairappan *et al.* 2009
(+)-elatol	*Ophionereis reticulata*	Paracuru Beach, Ceará State, Brazil (3º S)	Atlantic	Ecological$^{\#}$	Sequestration and bioaccumulation				Nuzzo *et al.* 2017

Pharmac= Pharmacological. # Chemical data available. † Stereochemistry not reported. * Identified in the extract; ** as *L. obtusa*. Miscel = Miscellaneous. LD= Lethal dose. IC= Inhibitory Concentration. CC= Cytotoxic Concentration. MIC= Minimum Inhibitory Concentration.
HM02: gastric carcinoma, HEP G2: liver carcinoma, MCF 7: breast carcinoma, HeLa: human carcinoma of the cervix, Hep-2: human carcinoma of the larynx, Vero: African green monkey kidney, P388: murine lymphocytic leukemia.

References

Arberas-Jiménez, I.; Nocchi, N.; Chao-Pellicer, J.; Sifaoui, I.; Soares, A.R.; Díaz-Marrero, A.R.; Fernández, J.J.; Piñero, J.E.; Lorenzo-Morales, J. Chamigrane-type sesquiterpenes from *Laurencia dendroidea* as lead compounds against *Naegleria fowleri*. *Mar. Drugs* **2023**, *21*, 224. https://doi.org/10.3390/MD21040224

Bansemir, A.; Just, N.; Michalik, M.; Lindequist, U.; Lalk, M. Extracts and sesquiterpene derivatives from the red alga *Laurencia chondrioides* with antibacterial activity against fish and human pathogenic bacteria. *Chem. Biodivers.* **2004**, *1*, 463–467. https://doi.org/10.1002/cbdv.200490039.

Barcellos Marini, M.; Rodrigues de Freitas, W.; da Silva Machado, F.L.; Correa Ramos Leal, I.; Ribeiro Soares, A.; Masahiko Kanashiro, M.; Frazão Muzitano, M. Cytotoxic activity of halogenated sesquiterpenes from *Laurencia dendroidea*. *Phytother. Res.* **2018**, *32*, 1119–1125. https://doi.org/10.1002/ptr.6052.

Biá Ventura, T.; da Silva Machado, F.; de Araujo, M.; de Souza Gestinari, L.; Kaiser, C.; Esteves, F.A.; Lasunskaia, E.; Soares, A.; Muzitano, M. Nitric oxide production inhibition and anti-mycobacterial activity of extracts and halogenated sesquiterpenes from the Brazilian red alga *Laurencia dendroidea* J. Agardh (Ceramiales: Rhodomelaceae). *Pharmacogn. Mag.* **2015**, *11*, 611. https://doi.org/10.4103/0973-1296.172972.

Bianco, É.M.; Krug, J.L.; Zimath, P.L.; Kröger, A.; Paganelli, C.J.; Boeder, A.M.; dos Santos, L.; Tenfen, A.; Ribeiro, S.M.; Kuroshima, K.N.; et al. Antimicrobial (including antimitocoplasmas), antioxidant and anticholinesterase activities of Brazilian and Spanish marine organisms – evaluation of extracts and pure compounds. *Braz. J. Pharmacogn.* **2015**, *25*, 668–676. https://doi.org/10.1016/j.bjp.2015.07.018.

Bianco, E.M.; Pires, L.; Santos, G.K.N.; Dutra, K.A.; Reis, T.N.V.; Vasconcelos, E.R.T.P.P.; Cocentino, A.L.M.; Navarro, D.M.A.F. Larvicidal activity of seaweeds from northeastern Brazil and of a halogenated sesquiterpene against the dengue mosquito (*Aedes aegypti*). *Ind. Crops Prod.* **2013**, *43*, 270–275. https://doi.org/10.1016/j.indcrop.2012.07.032.

Born, F.S.; Bianco, É.M.; da Camara, C.A.G. Acaricidal and repellent activity of terpenoids from seaweeds collected in Pernambuco, Brazil. *Nat. Prod. Commun.* **2012**, *7*, 463–466.

Brennan, M.R.; Erickson, K.L.; Minott, D.A.; Pascoe, K.O. Chamigrane metabolites from a Jamaican variety of *Laurencia obtusa*. *Phytochemistry* **1987**, *26*, 1053–1057. https://doi.org/10.1016/S0031-9422(00)82349-3.

Campos, A.; Souza, C.B.; Lhullier, C.; Falkenberg, M.; Schenkel, E.P.; Ribeiro-Do-Valle, R.M.; Siqueira, J.M. Anti-tumour effects of elatol, a marine derivative compound obtained from red algae *Laurencia microcladia*. *J. Pharm. Pharmacol.* **2012**, *64*, 1146–1154. https://doi.org/10.1111/j.2042-7158.2012.01493.x.

Capon, R.J.; Ghisalberti, E.L.; Mori, T.A.; Jefferies, P.R. Sesquiterpenes from *Laurencia* spp. *J. Nat. Prod.* **1988**, *51*, 1302–1304. https://doi.org/10.1021/np50060a049.

Chao-Pellicer, J.; Arberas-Jiménez, I.; Sifaoui, I.; Díaz-Marrero, A.R.; Fernández, J.J.; Jamerson, M.; Piñero, J.E.; Lorenzo-Morales, J. Potential inhibitors of human–*Naegleria fowleri* interactions: An in vitro extracellular matrix-based model. *Mar. Drugs* **2025**, *23*, 306. https://doi.org/10.3390/MD23080306.

Cunningham, T.A.; Maiti, P.; Manara, P.; Singh, V.; Beaton, N.; Amunts, A.; Fontanesi, F.; Barrientos, A.; Schatz, J.H. The marine product elatol is a novel inhibitor of mitochondrial translation triggering the integrated stress response and apoptosis in leukemia and lymphoma cells. *Blood* **2022**, *140*, 7819. https://doi.org/10.1182/BLOOD-2022-166899.

Da Gama, B.A.P.; Pereira, R.C.; Soares, A.R.; Teixeira, V.L.; Yoneshigue-Valentin, Y. Is the mussel test a good indicator of antifouling activity? A comparison between laboratory and field assays. *Biofouling* **2003**, *19*, 161–169. https://doi.org/10.1080/0892701031000089534.

De Nys, R.; Leya, T.; Maximilien, R.; Afsar, A.; Nair, P.S.R.; Steinberg, P.D. The need for standardised broad scale bioassay testing: A case study using the red alga *Laurencia rigida*. *Biofouling* **1996**, *10*, 213–224. https://doi.org/10.1080/08927019609386281.

Desoti, V.C.; Lazarin-Bidóia, D.; Bueno Sudatti, D.; Crespo Pereira, R.; Ueda-Nakamura, T.; Vataru Nakamura, C.V.; de Oliveira Silva, S. Additional evidence of the trypanocidal action of (–)-elatol on amastigote forms through the involvement of reactive oxygen species. *Mar. Drugs* **2014**, *12*, 4973–4983. https://doi.org/10.3390/md12094973.

Desoti, V.C.; Lazarin-Bidóia, D.; Sudatti, D.B.; Pereira, R.C.; Alonso, A.; Ueda-Nakamura, T.; Dias Filho, B.P.; Nakamura, C.V.; de Silva, S.O. Trypanocidal action of (–)-elatol involves an oxidative stress triggered by mitochondria dysfunction. *Mar. Drugs* **2012**, *10*, 1631–1646. https://doi.org/10.3390/md10081631.

Dias, T.; Brito, I.; Moujir, L.; Paiz, N.; Darias, J.; Cueto, M. Cytotoxic sesquiterpenes from *Aplysia dactylomela*. *J. Nat. Prod.* **2005**, *68*, 1677–1679. https://doi.org/10.1021/np050240y.

Díaz-Marrero, A.R.; Brito, I.; de la Rosa, J.M.; D'Croz, L.; Fabelo, O.; Ruiz-Pérez, C.; Cueto, M. Novel lactone chamigrene-derived metabolites from *Laurencia majuscula*. *European Journal of Organic Chemistry* **2009**, 2009, 5037–5043. https://doi.org/10.1002/ejoc.200801006.

Díaz-Marrero, A.R.; de la Rosa, J.M.; Brito, I.; Darias, J.; Cueto, M. Dactylomelatriol, a biogenetically intriguing omphalane-derived marine sesquiterpene. *Journal of Natural Products* **2012**, *75*, 866–869. https://doi.org/10.1021/np200845f.

Dos Santos, A.O.; Veiga-Santos, P.; Ueda-Nakamura, T.; Dias Filho, B.P.; Sudatti, D.B.; Bianco, É.M.; Pereira, R.C.; Nakamura, C.V. Effect of elatol, isolated from red seaweed *Laurencia dendroidea*, on *Leishmania amazonensis*. *Mar. Drugs* **2010**, *8*, 2733–2743. https://doi.org/10.3390/md8112733.

Dos Santos, G.S.; Miyasato, P.A.; Stein, E.M.; Colepicolo, P.; Wright, A.D.; Pereira, C.A. de B.; Falkenberg, M.; Nakano, E. Algal-derived halogenated sesquiterpenes from *Laurencia dendroidea* as lead compounds in schistosomiasis environmental control. *Mar. Drugs* **2022**, *20*, 111. https://doi.org/10.3390/md20020111.

Fenical, W.; Norris, J.N. Chemotaxonomy in marine algae: Chemical separation of some *Laurencia* species (Rhodophyta) from the Gulf of California. *J. Phycol.* **1975**, *11*, 104–108.

Fernandes Peres, J.C.; Retz de Carvalho, L.; Gonçalez, E.; Otávio, L.; Berian, S.; Felicio, J.D.; Cesar, J.; Peres, F.; de Carvalho, L.R.; Gonçalez, E.; et al. Evaluation of antifungal activity of seaweed extracts. *Cienc. Agrotecnol.* **2012**, *36*, 294–299.

Gerwick, W.H.; Lopez, A. Two new chamigrene sesquiterpenoids from the tropical red alga *Laurencia obtusa*. *J. Nat. Prod.* **1987**, *50*, 1131–1135. https://doi.org/10.1021/np50054a020.

Gonçalves, K.G.; da Silva, L.L.; Soares, A.R.; Romeiro, N.C. Acetylcholinesterase as a target of halogenated marine natural products from *Laurencia dendroidea*. *Algal Res.* **2020**, *52*, 102130. https://doi.org/10.1016/j.algal.2020.102130.

González, A.G.; Darias, J.; Díaz, A.; Fourneron, J.D.; Martín, J.D.; Pérez, C. Evidence for the biogenesis of halogenated chamigrenes from the red alga *Laurencia obtusa*. *Tetrahedron Lett.* **1976**, *17*, 3051–3054. https://doi.org/10.1016/0040-4039(76)80067-6.

Granado, I.; Caballero Ortega, P. Chemical defense in the seaweed *Laurencia obtusa* (Hudson) Lamouroux. *Sci. Mar.* **1995**, *59*, 31–39.

Hay, M.E.; Fenical, W.; Gustafson, K. Chemical defense against diverse coral-reef herbivores. *Ecology* **1987**, *68*, 1581–1591.

Ji, N.Y.; Li, X.M.; Cui, C.M.; Wang, B.G. Terpenes and polybromoindoles from the marine red alga *Laurencia decumbens* (Rhodomelaceae). *Helvetica Chimica Acta* **2007a**, *90*, 1731–1736. https://doi.org/10.1002/hlca.200790181.

Ji, N.Y.; Li, X.M.; Li, K.; Ding, L.P.; Gloer, J.B.; Wang, B.G. Diterpenes, sesquiterpenes, and a C15-acetogenin from the marine red alga *Laurencia mariannensis*. *Journal of Natural Products* **2007b**, *70*, 1901–1905. https://doi.org/10.1021/np070378b.

Ji, N.Y.; Li, X.M.; Ding, L.P.; Wang, B.G. Aristolane sesquiterpenes and highly brominated indoles from the marine red alga *Laurencia similis* (Rhodomelaceae). *Helvetica Chimica Acta* **2007c**, *90*, 385–391. https://doi.org/10.1002/hlca.200790044.

Jiménez-Romero, C.; Mayer, A.M.S.; Rodríguez, A.D. Dactyloditerpenol acetate, a new prenylbisabolane-type diterpene from *Aplysia dactylomela* with significant in vitro anti-neuroinflammatory activity. *Bioorg. Med. Chem. Lett.* **2014**, *24*, 344–348. https://doi.org/10.1016/j.bmcl.2013.11.008.

Juagdan, E.G.; Kalidindi, R.; Scheuer, P. Two new chamigranes from an Hawaiian red alga, *Laurencia cartilaginea*. *Tetrahedron* **1997**, *53*, 521–528. https://doi.org/10.1016/S0040-4020(96)01002-2.

Kamada, T.; Phan, C.-S.; Okino, T.; Vairappan, C.S. Cytotoxicity and antibacterial potential of halogenated chamigrenes from Malaysian red alga, *Laurencia majuscula*. *Planta Med. Int. Open* **2019**, *6*, e36–e40. https://doi.org/10.1055/a-0977-4418.

Kamada, T.; Phan, C.S.; Vairappan, C.S. New anti-bacterial halogenated tricyclic sesquiterpenes from Bornean *Laurencia majuscula* (Harvey) Lucas. *Nat. Prod. Res.* **2017**, *33*, 464–471. https://doi.org/10.1080/14786419.2017.1396593.

Kennedy, D.J.; Selby, I.A.; Thomson, R.H. Chamigrane metabolites from *Laurencia obtusa* and *L. scoparia*. *Phytochemistry* **1988**, *27*, 1761–1766. https://doi.org/10.1016/0031-9422(88)80439-4.

König, G.M.; Wright, A.D. *Laurencia rigida*: Chemical investigations of its antifouling dichloromethane extract. *J. Nat. Prod.* **1997**, *60*, 967–970. https://doi.org/10.1021/NP970181R.

Lang, K.L.; Silva, I.T.; Zimmermann, L.A.; Lhullier, C.; Arana, M.V.M.; Palermo, J.A.; Falkenberg, M.; Simões, C.M.O.; Schenkel, E.P.; Durán, F.J. Cytotoxic activity of semi-synthetic derivatives of elatol and isoobtusol. *Mar. Drugs* **2012**, *10*, 2254–2264. https://doi.org/10.3390/md10102254.

Lhullier, C.; Donnangelo, A.; Caro, M.; Palermo, J.A.; Horta, P.A.; Falkenberg, M.; Schenkel, E.P. Isolation of elatol from *Laurencia microcladia* and its palatability to the sea urchin *Echinometra lucunter*. *Biochem. Syst. Ecol.* **2009**, *37*, 254–259. https://doi.org/10.1016/j.bse.2009.04.004.

Machado, F.L.; Pacienza-Lima, W.; Rossi-Bergmann, B.; de Souza Gestinari, L.M.; Fujii, M.T.; Campos de Paula, J.; Costa, S.S.; Lopes, N.P.; Kaiser, C.R.; Soares, A.R. Antileishmanial sesquiterpenes from the Brazilian red alga *Laurencia dendroidea* J. Agardh. *Planta Med.* **2011**, *77*, 733–735. https://doi.org/10.1055/s-0030-1250526.

Machado, F.L.S.; Duarte, H.M.; Gestinari, L.M.S.; Cassano, V.; Kaiser, C.R.; Soares, A.R. Geographic distribution of natural products produced by the red alga *Laurencia dendroidea* J. Agardh. *Chem. Biodivers.* **2016**, 845–851. https://doi.org/10.1002/cbdv.201500246.

Machado, F.L.S.; Lima, W.P.; Duarte, H.M.; Rossi-Bergmann, B.; Gestinari, L.M.; Fujii, M.T.; Kaiser, C.R.; Soares, A.R. Chemical diversity and antileishmanial activity of crude extracts of the *Laurencia* complex (Ceramiales, Rhodophyta) from Brazil. *Braz. J. Pharmacogn.* **2014**, *24*, 635–643. https://doi.org/10.1016/j.bjp.2014.10.009.

Maiti, P.; Cunningham, T.A.; Barrientos, A.; Schatz, J.H. Inhibition of mitochondrial translation by the marine natural product elatol shows potent antileukemia activity. *Blood* **2021**, *138*, 4342. https://doi.org/10.1182/blood-2021-153347.

Martin, J.D.; Caballero, P.; Fernandez, J.J.; Norte, M.; Perez, R.; Rodríguez, M.L. Metabolites from *Laurencia obtusa*. *Phytochemistry* **1989**, *28*, 3365–3367. https://doi.org/10.1016/0031-9422(89)80348-6.

Masuda, M.; Kogame, K.; Arisawa, S.; Suzuki, M. Morphology and halogenated secondary metabolites of three Gran Canarian species of *Laurencia* (Ceramiales, Rhodophyta). *Botanica Marina* **1998**, *41*, 265–272. https://doi.org/10.1515/botm.1998.41.1-6.265.

Nag, A.; Banerjee, R.; Chowdhury, R.R.; Krishnapura Venkatesh, C. Phytochemicals as potential drug candidates for targeting SARS-CoV-2 proteins, an in silico study. *Virusdisease* **2021**, *32*, 98–107. https://doi.org/10.1007/S13337-021-00654-X.

Nocchi, N.; Soares, A.R.; Souto, M.L.; Fernández, J.J.; Martin, M.N.; Pereira, R.C. Detection of a chemical cue from the host seaweed *Laurencia dendroidea* by the associated mollusc *Aplysia brasiliana*. *PLoS ONE* **2017**, *12*, e0187126. https://doi.org/10.1371/journal.pone.0187126.

Norris, J.N.; Fenical, W. Chemical defense in tropical marine algae. The Atlantic Barrier Reef Ecosystem at Carrie Bow Cay, Belize. *Prof. Pap. Mar. Sci.* **1982**, *12*, 417–431.

Nuzzo, G.; Gomes, B.A.; Amodeo, P.; Matthews-Cascon, H.; Cutignano, A.; Costa-Lotufo, L.V.; Monteiro, F.A.C.; Pessoa, O.D.L.; Fontana, A. Isolation of chamigrene sesquiterpenes and absolute configuration of isoobtusadiene from the brittle star *Ophionereis reticulata*. *J. Nat. Prod.* **2017**, *80*, 3049–3053. https://doi.org/10.1021/acs.jnatprod.7b00510.

Oliveira, A.S.; Sudatti, D.B.; Fujii, M.T.; Rodrigues, S.V.; Pereira, R.C. Inter- and intrapopulation variation in the defensive chemistry of the red seaweed *Laurencia dendroidea* (Ceramiales, Rhodophyta). *Phycologia* **2013**, *52*, 130–136.

Palaniveloo, K.; Vairappan, C.S. Chemical relationship between red algae genus *Laurencia* and sea hare (*Aplysia dactylomela* Rang) in the North Borneo Island. *J. Appl. Phycol.* **2014**, *26*, 1199–1205. https://doi.org/10.1007/s10811-013-0127-z.

Paradas, W.C.; Salgado, L.T.; Sudatti, D.B.; Crapez, M.A.; Fujii, M.T.; Coutinho, R.; Pereira, R.C.; Amado Filho, G.M. Induction of halogenated vesicle transport in cells of the red seaweed *Laurencia obtusa*. *Biofouling* **2010**, *26*, 277–286. https://doi.org/10.1080/08927010903515122.

Pereira, R.C.; Da Gama, B.A.; Teixeira, V.L.; Yoneshigue-Valentin, Y. Ecological roles of natural products of the Brazilian red seaweed *Laurencia obtusa*. *Braz. J. Biol.* **2003**, *63*, 665–672. https://doi.org/10.1590/S1519-69842003000400013.

Pereira, R.C.; Nocchi, N.; Souto, M.L.; Fernández, J.J.; Norte, M.; Duarte, H.M.; Soares, A.R. The sea-hare *Aplysia brasiliana* promotes induction in chemical defense in the seaweed *Laurencia dendroidea* and in their congeneric neighbors. *Plant Physiol. Biochem.* **2020**, *154*, 295–303. https://doi.org/10.1016/j.plaphy.2020.05.020.

Peters, T.L.; Tillotson, J.; Yeomans, A.M.; Wilmore, S.; Lemm, E.; Jimenez-Romero, C.; Amador, L.A.; Li, L.; Amin, A.D.; Pongtornpipat, P.; et al. Target-based screening against EIF4A1 reveals the marine natural product elatol as a novel inhibitor of translation initiation with in vivo antitumor activity. *Clin. Cancer Res.* **2018**, *24*, 4256–4270. https://doi.org/10.1158/1078-0432.CCR-17-3645.

Pokharkar, O.; Anumolu, H.; Zyryanov, G.V.; Tsurkan, M.V. Natural products from red algal genus *Laurencia* as potential inhibitors of RdRp and Nsp15 enzymes of SARS-CoV-2: An *in silico* perspective. *Microbiol. Res. (Pavia)* **2023**, *14*, 1020–1048. https://doi.org/10.3390/MICROBIOLRES14030069.

Rose, A.F.; Sims, J.J. A metabolite of the marine alga *Laurencia* sp. Pergamon Press, **1977**, *34*.

Salgado, L.T.; Oliveira, L.S.; Echevarria-Lima, J.; Reis, V.M.; Sudatti, D.B.; Thompson, F.L. Role of ABC proteins in secondary metabolism and immune (=defensive) response in seaweeds. *Cells* **2023**, *12*, 2259.

Salgado, L.T.; Viana, N.B.; Andrade, L.R.; Leal, R.N.; da Gama, B.A.P.; Attias, M.; Pereira, R.C.; Amado Filho, G.M. Intra-cellular storage, transport and exocytosis of halogenated compounds in marine red alga *Laurencia obtusa*. *J. Struct. Biol.* **2008**, *162*, 345–355. https://doi.org/10.1016/j.jsb.2008.01.015.

Salvador-Neto, O.; Gomes, S.A.; Soares, A.R.; da Silva Machado, F.L.; Samuels, R.I.; de Souza-Menezes, J.; da Cunha Moraes, J.L.; Campos, E.; Mury, F.B.; et al. Larvicidal potential of the halogenated sesquiterpene (+)-obtusol, isolated from the alga *Laurencia dendroidea* J. Agardh (Ceramiales: Rhodomelaceae), against the dengue vector mosquito *Aedes aegypti* (Linnaeus) (Diptera: Culicidae). *Mar. Drugs* **2016**, *14*, 20. https://doi.org/10.3390/md14020020.

Schmitz, F.J.; Michaud, D.P.; Schmidt, P.G. Marine natural products: parguerol, deoxyparguerol, and isoparguerol. New brominated diterpenes with modified pimarane skeletons from the sea hare *Aplysia dactylomela*. *Isr. J. Chem.* **1982**, *104*, 281.

Sims, J.J.; Lin, G.H.Y.; Wing, R.M. Marine natural products X: elatol, a halogenated sesquiterpene alcohol from the red alga *Laurencia elata*. *Tetrahedron Lett.* **1974**, *15*, 3487–3490. https://doi.org/10.1016/S0040-4039(01)91944-6.

Soares, A.R.; Robaina, M.C.S.S.; Mendes, G.S.; Silva, T.S.L.L.; Gestinari, L.M.S.S.; Pamplona, O.S.; Yoneshigue-Valentin, Y.; Kaiser, C.R.; Romanos, M.T.V.; Teresa, M.; et al. Antiviral activity of extracts from Brazilian seaweeds against herpes simplex virus. *Braz. J. Pharmacogn.* **2012**, *22*, 714–723.

Sudatti, D.B.; Duarte, H.M.; Soares, A.R.; Salgado, L.T.; Pereira, R.C. New ecological role of seaweed secondary metabolites as autotoxic and allelopathic. *Front. Plant Sci.* **2020**, *11*, 347. https://doi.org/10.3389/fpls.2020.00347.

Sudatti, D.B.; Fujii, M.T.; Rodrigues, S.V.; Turra, A.; Duarte, H.M.; Soares, A.R.; Pereira, R.C. Diel variation of sesquiterpene elatol production: A chemical defense mechanism of the red seaweed *Laurencia dendroidea*. *Biochem. Syst. Ecol.* **2016**, *64*, 131–135. https://doi.org/10.1016/j.bse.2015.12.001.

Sudatti, D.B.; Fujii, M.T.; Rodrigues, S.V.; Turra, A.; Pereira, R.C. Effects of abiotic factors on growth and chemical defenses in cultivated clones of *Laurencia dendroidea* J. Agardh (Ceramiales, Rhodophyta). *Mar. Biol.* **2011**, *158*, 1439–1446. https://doi.org/10.1007/s00227-011-1660-4.

Sudatti, D.B.; Fujii, M.T.; Rodrigues, S.V.; Turra, A.; Pereira, R.C. Prompt induction of chemical defenses in the red seaweed *Laurencia dendroidea*: The role of herbivory and epibiosis. *Journal of Sea Research* **2018**, *138*, 48–55. https://doi.org/10.1016/j.seares.2018.04.007.

Sudatti, D.B.; Oliveira, A.S.; da Gama, B.A.P.; Fujii, M.T.; Rodrigues, S.V.; Pereira, R.C. Variability in seaweed chemical defense and growth under common garden conditions. *Frontiers in Marine Science* **2021**, *8*, 720711. https://doi.org/10.3389/fmars.2021.720711.

Sudatti, D.B.; Rodrigues, S.V.; Coutinho, R.; da Gama, B.A.P.; Salgado, L.T.; Amado Filho, G.M.; Pereira, R.C. Transport and defensive role of elatol at the surface of the red seaweed *Laurencia obtusa* (Ceramiales, Rhodophyta). *J. Phycol.* **2008**, *44*, 584–591. https://doi.org/10.1111/j.1529-8817.2008.00507.x.

Sudatti, D.B.; Rodrigues, S.V.; Pereira, R.C. Quantitative GC-ECD analysis of halogenated metabolites: Determination of surface and within-thallus elatol of *Laurencia obtusa*. *J. Chem. Ecol.* **2006**, *32*, 835–843. https://doi.org/10.1007/s10886-006-9033-z.

Vairappan, C.S. Potent antibacterial activity of halogenated metabolites from Malaysian red algae, *Laurencia majuscula* (Rhodomelaceae, Ceramiales). *Biomol. Eng.* **2003**, *20*, 255–259. https://doi.org/10.1016/S1389-0344(03)00067-4.

Vairappan, C.S.; Anangdan, S.P.; Matsunaga, S. Diet-derived halogenated metabolite from the sea hare *Aplysia parvula*. *Malaysian J. Sci.* **2009**, *28*, 269–273.

Vairappan, C.S.; Anangdan, S.P.; Tan, K.L.; Matsunaga, S. Role of secondary metabolites as defense chemicals against ice-ice disease bacteria in biofouler at carrageenophyte farms. *J. Appl. Phycol.* **2010**, *22*, 305–311. https://doi.org/10.1007/s10811-009-9460-7.

Vairappan, C.S.; Daitoh, M.; Suzuki, M.; Abe, T.; Masuda, M. Antibacterial halogenated metabolites from the Malaysian *Laurencia* species. *Phytochemistry* **2001**, *58*, 291–297. https://doi.org/10.1016/S0031-9422(01)00243-6.

Vairappan, C.S.; Phang, P.S. Morphology and halochamigrene metabolite content of *Laurencia majuscula* (Rhodomelaceae, Ceramiales) from the Spratly Islands. *Malaysian Journal of Science* **2005**, *24*, 29–36.

Veiga-Santos, P.; Pelizzaro-Rocha, K.J.; Santos, A.O.; Ueda-Nakamura, T.; Filho, B.P.D.; Silva, S.O.; Sudatti, D.B.; Bianco, E.M.; Pereira, R.C.; Nakamura, C.V. In vitro anti-trypanosomal activity of elatol isolated from red seaweed *Laurencia dendroidea*. *Parasitology* **2010**, *137*, 1661–1670. https://doi.org/10.1017/S003118201000034X.

Vera, B.; Rodríguez, A.D.; Avilés, E.; Ishikawa, Y. Aplysqualenols A and B: Squalene-derived polyethers with antitumoral and antiviral activity from the Caribbean sea slug *Aplysia dactylomela*. *Eur. J. Org. Chem.* **2009**, 5327–5336. https://doi.org/10.1002/ejoc.200900775.

Wall, M.E. Antimutagenic agents from natural products. *J. Nat. Prod.* **1992**, *55*, 1561–1566. https://doi.org/10.1021/NP50089A002.

Wessels, M.; König, G.M.; Wright, A.D. New natural product isolation and comparison of the secondary metabolite content of three distinct samples of the sea hare *Aplysia dactylomela* from Tenerife. *J. Nat. Prod.* **2000**, *63*, 920–928. https://doi.org/10.1021/np9905721.
